# Supplementary material for: Interplay between coding and non-coding regulation drives the Arabidopsis seed-to-seedling transition
Source: Nat Commun. 2024 Feb 26;15:1724. doi: 10.1038/s41467-024-46082-5 (PMC10897432; doi:10.1038/s41467-024-46082-5)
Supplement: Supplementary file 1 — Supplementary Information [file 41467_2024_46082_MOESM1_ESM.pdf]

Supplementary information

Interplay between coding and non-coding regulation drives the Arabidopsis seed-to-seedling transition

Benjamin J.M. Tremblay, Cristina P. Santini, Yajiao Cheng, Xue Zhang, Stefanie Rosa, Julia I. Qüesta

Supplementary Figures 1-10

Supplementary Tables 1-3

Supplementary Note 1

Supplementary Figures 11-13

Supplementary Note 2

Supplementary Figures 14-15

Supplementary References

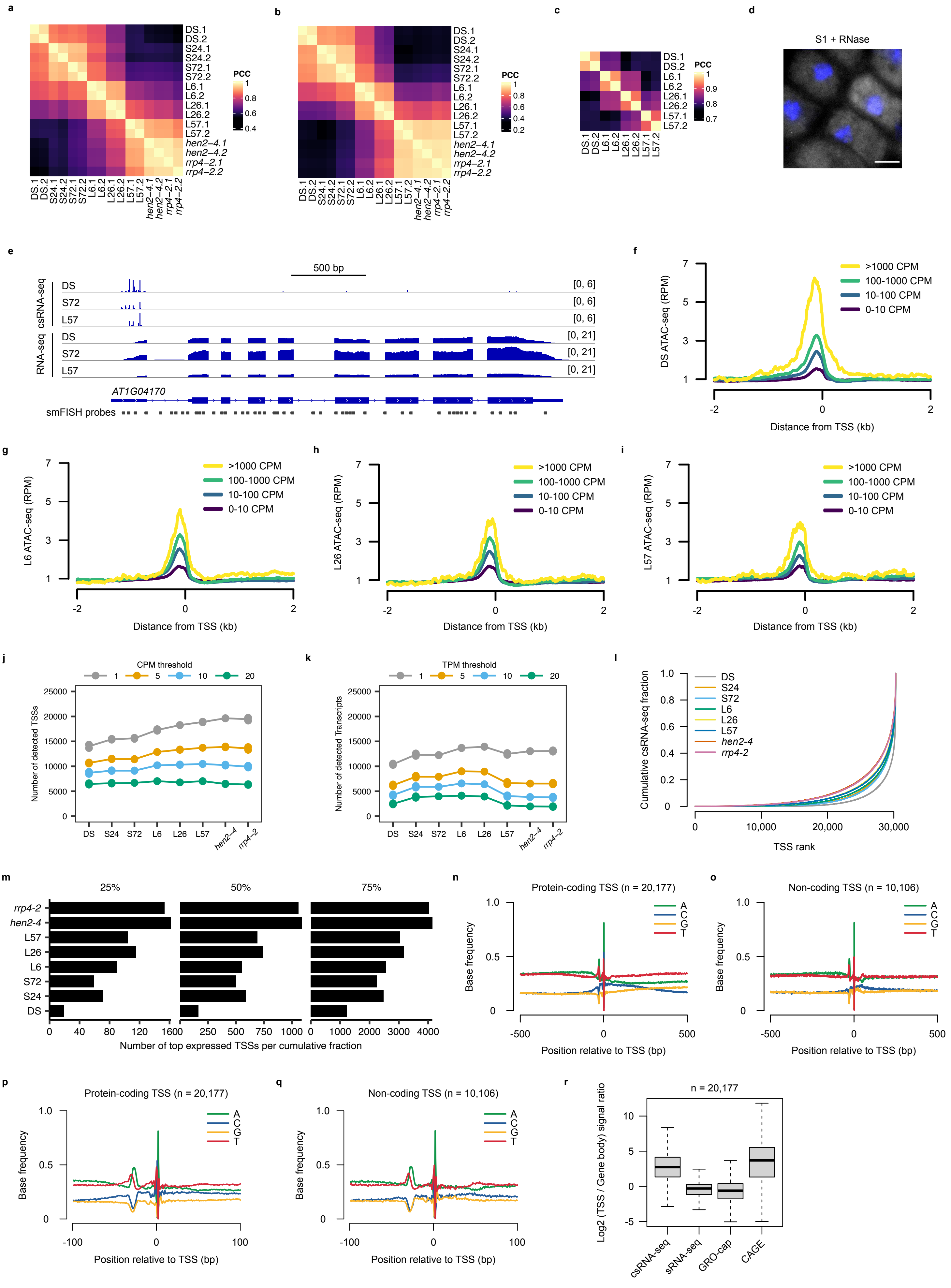

Supplementary Figure 1: The csRNA-seq provides a more accurate view of transcription events during germination.

(a) Pearson correlation coefficient heatmap of normalized csRNA-seq expression at detected TSSs across all samples.

(b) Same as (a) for the RNA-seq, using normalized transcription quantification data.

(c) Same as (a) for the ATAC-seq, using normalized ACR quantification data.

(d) RNase control of smFISH in 1 h imbibed seeds (S1) using probes for the unspliced RNA of *AT1G04170*. The scale bar represents 10 µm. Experiments were repeated independently at least two times.

(e) csRNA-seq and RNA-seq coverage tracks at a few sample time-points (DS, S72, L57) for the gene *AT1G04170* (units in RPM). Also shown are the smFISH probes used in Figure 1d.

(f), (g), (h) and (i) ATAC-seq read density in the 4 Kbp region around detected TSSs. The signal is split into four based on the expression brackets of the TSSs in the matching csRNA-seq samples corresponding to the DS, L6, L26 and L57 ATAC-seq samples.

(j) Number of detected TSSs with matching CPM thresholds per sample.

(k) Number of detected genes with matching TPM thresholds per sample (using the highest expressing isoform as the gene representative).

(l) Cumulative csRNA-seq signal of all detected TSSs in all samples, ordered along the x-axis by their expression level (most expressed last).

(m) Number of TSSs per csRNA-seq sample within the top 25%, 50% and 75% expressed TSSs.

(n) Promoter base composition in a 1 Kbp region around all detected protein coding TSSs.

(o) Same as (m) for all detected non-coding TSSs.

(p) Same as (m), for a smaller 200 bp region around the TSSs.

(q) Same as (n), for a smaller 200 bp region around the TSSs.

(r) The $\log_2$ ratio of TSS signal (in a 200 bp window centered around each TSS) to gene body signal (in a 1 Kbp region 200 bp downstream of the TSS) for the L57 csRNA-seq and sRNA-seq, in comparison with GRO-cap (Hetzel et al., 2016) and CAGE (Thieffry et al., 2020) samples. The lower, middle and upper hinges correspond to first quartile, median, and third quartile, respectively. The lower and upper whiskers extend to the minimal/maximal value respectively or 1.5 times the interquartile range, whichever is closer to the median.

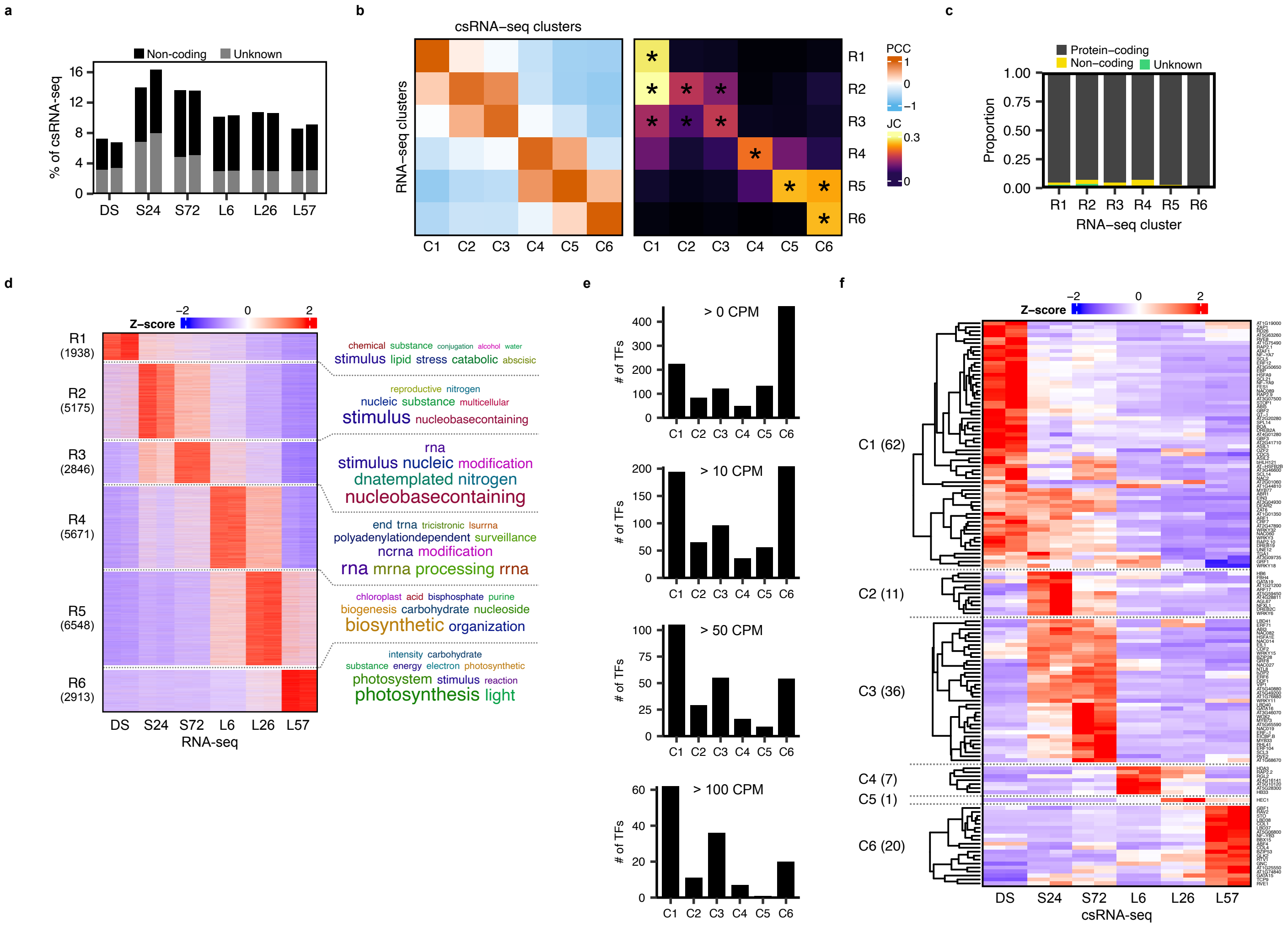

Supplementary Figure 2: Additional functional gene programs during germination.

(a) Percent of all reads in detected TSSs per csRNA-seq sample being in TSSs annotated as putative lncRNA (non-coding) or to no transcript (unknown).

(b) Comparison analyses of the RNA-seq and csRNA-seq clusters. The left heatmap shows the Pearson correlation coefficient between the average Z-score profiles of each cluster. The right heatmap shows the Jaccard coefficient of the number of common associated genes of each cluster. Comparisons with significant overlap are marked with * (P-value < 10^{-6}). Significance testing was performed using Fisher's exact test without correction for multiple testing.

(c) Proportion of annotated transcript types in the RNA-seq clusters. All non-protein coding Araport11 transcripts are labeled as non-coding, and putative reconstructed lncRNAs as unknown.

(d) Heatmap of developmental clusters from the RNA-seq time-series. Rows represent z-scores of the expression of individual transcript. Associated genes were enriched for overrepresented gene ontology terms, followed by an individual keyword enrichment analysis to generate word clouds of overrepresented keywords to the right of the heatmap, with their size being proportional to the level of enrichment.

(e) Bar plots for the number of transcription factors associated with each csRNA-seq cluster passing various thresholds of expression (taken from the sample associated with each cluster).

(f) Heatmap of expression of high expressing TSSs (>100 CPM in the sample associated with each cluster) from the csRNA-seq clusters associated with transcription factor genes.

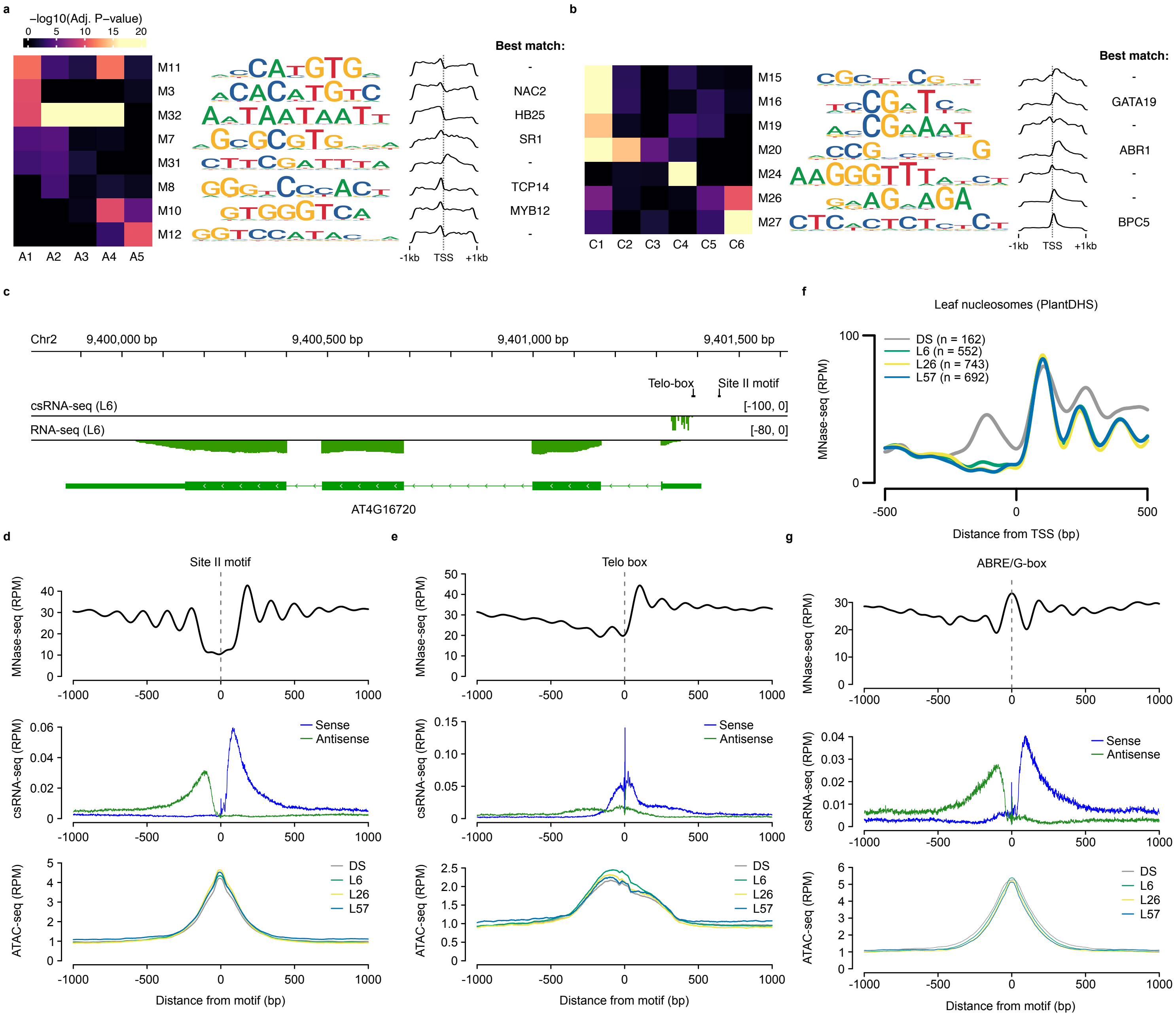

Supplementary Figure 3: Additional properties of enriched transcription factor binding sites.

(a) Enrichment of discovered motifs from the ACRs found in the ATAC-seq clusters but not the promoters of TSSs found in the csRNA-seq clusters. A heatmap shows the level of enrichment (-$\log_{10}$P-value) of the motif in each cluster, with each row representing a unique motif (shown to the right as an information content motif logo). The density of the motifs is shown to demonstrate their positional preference in promoters. The best matching known binding transcription and/or element name is included on the right. P-values were calculated using one-sided Fisher's exact tests with FDR correction for multiple testing.

(b) Same as (a) for discovered motifs from the promoters of TSSs found in the csRNA-seq clusters and not the ACRs found in the ATAC-seq clusters.

(c) csRNA-seq and RNA-seq coverage tracks for an example ribosomal gene (*AT4G16720*); with both a Telo-box and a Site II motif in its promoter. Units are in RPM.

(d) Average leaf MNase-seq (Zhang et al., 2016), csRNA-seq (merged from all samples) and ATAC-seq signal in a 2 Kbp region centered around M2 motifs (Site II) found in ACRs and TSS promoters.

(e) Same as (d) for the M23 motif (Telo-box).

(f) Average leaf MNase-seq (Zhang et al., 2016) signal in a 1 Kbp region centered at the top expressed TSSs (top 50% cumulative fraction) from the DS, L6, L26 and L57 samples.

(g) Same as (d) for the M1 motif (ABRE/G-box).

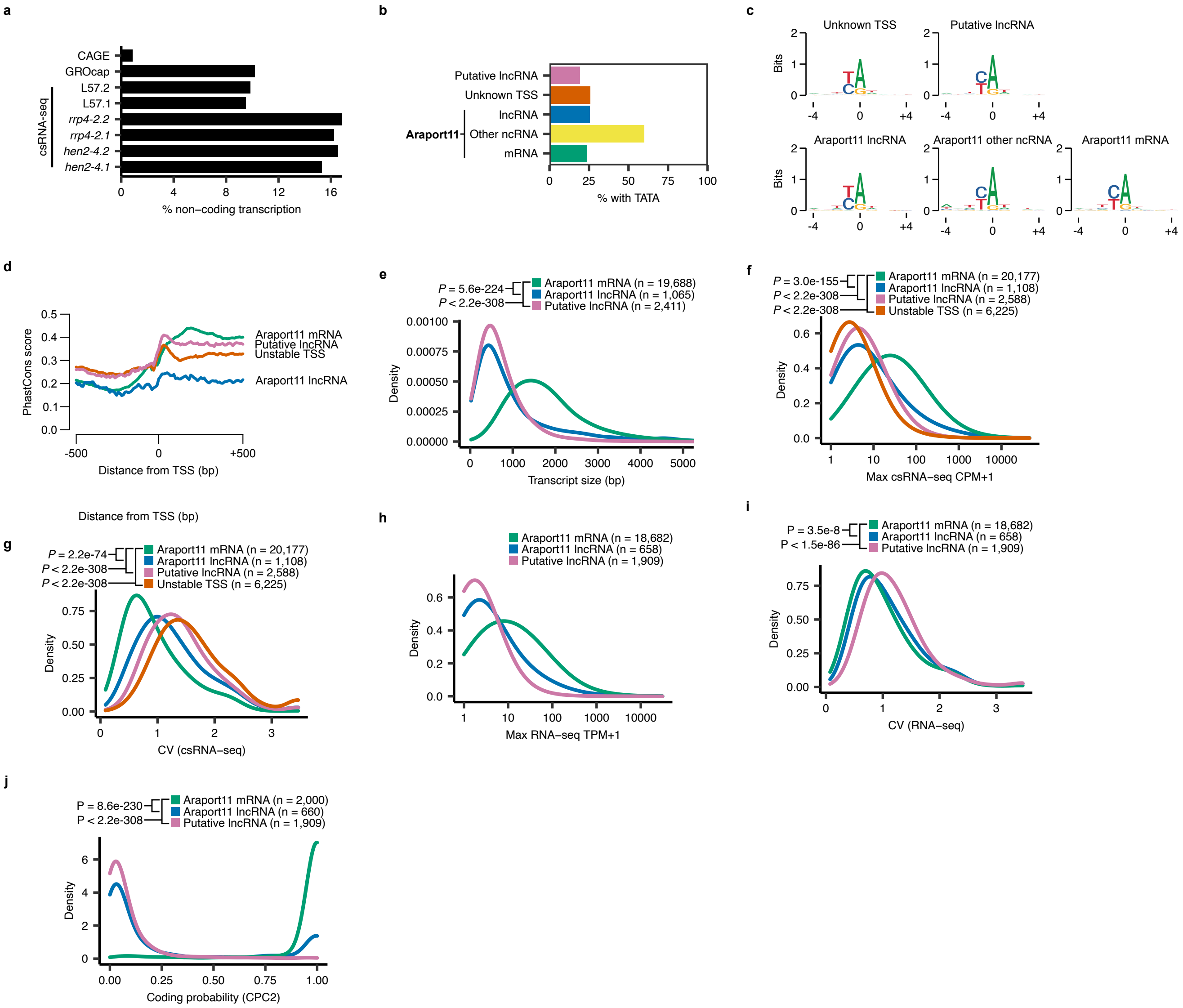

Supplementary Figure 4: Additional properties of non-coding transcription initiation from the csRNA-seq.

(a) Percent of all reads in detected TSSs for the L57, *hen2-4* and *rrp4-2* csRNA-seq samples, as well as the GRO-cap (Hetzel et al., 2016) and CAGE (Thieffry et al., 2020) samples being in TSSs not annotated as protein coding.

(b) Percent of TSSs by annotation type with a detected TATA box.

(c) Inr element motifs for TSSs by annotation type. The motifs are plotted as information content matrices taken from the peak of each TSS in the annotation classes.

(d) Average conservation of promoters by annotated TSS type (mRNA, lncRNA, Putative lncRNA, and unstable TSS), using PhastCons scores calculated from 63 plants (Tian et al., 2020). The coverage of scores is from 500 bp upstream and downstream of the primary TSS coordinate.

(e) Density plot of transcript sizes for Araport11 protein coding genes, lncRNAs, and putative lncRNAs.

(f) Density plot of the max expression (on a $\log_{10}$ scale) of each annotated TSS type across all sampled csRNA-seq time-points.

(g) Density plot for the coefficient of variation of the csRNA-seq quantification data for each annotated TSS type.

(h) Density plot of the max expression (on a $\log_{10}$ scale) of each annotated transcript type across all sampled RNA-seq time-points.

(i) Density plot for the coefficient of variation of the RNA-seq quantification data for each annotated transcript type. Significance testing between annotation types for (e), (f), (g), and (i) was performed using two-sided Mann-Whitney tests with Holm correction for multiple testing.

(j) Density plot of the coding probability of each annotated transcript type calculated using the Coding Potential Calculator (CPC2; Kang et al., 2017).

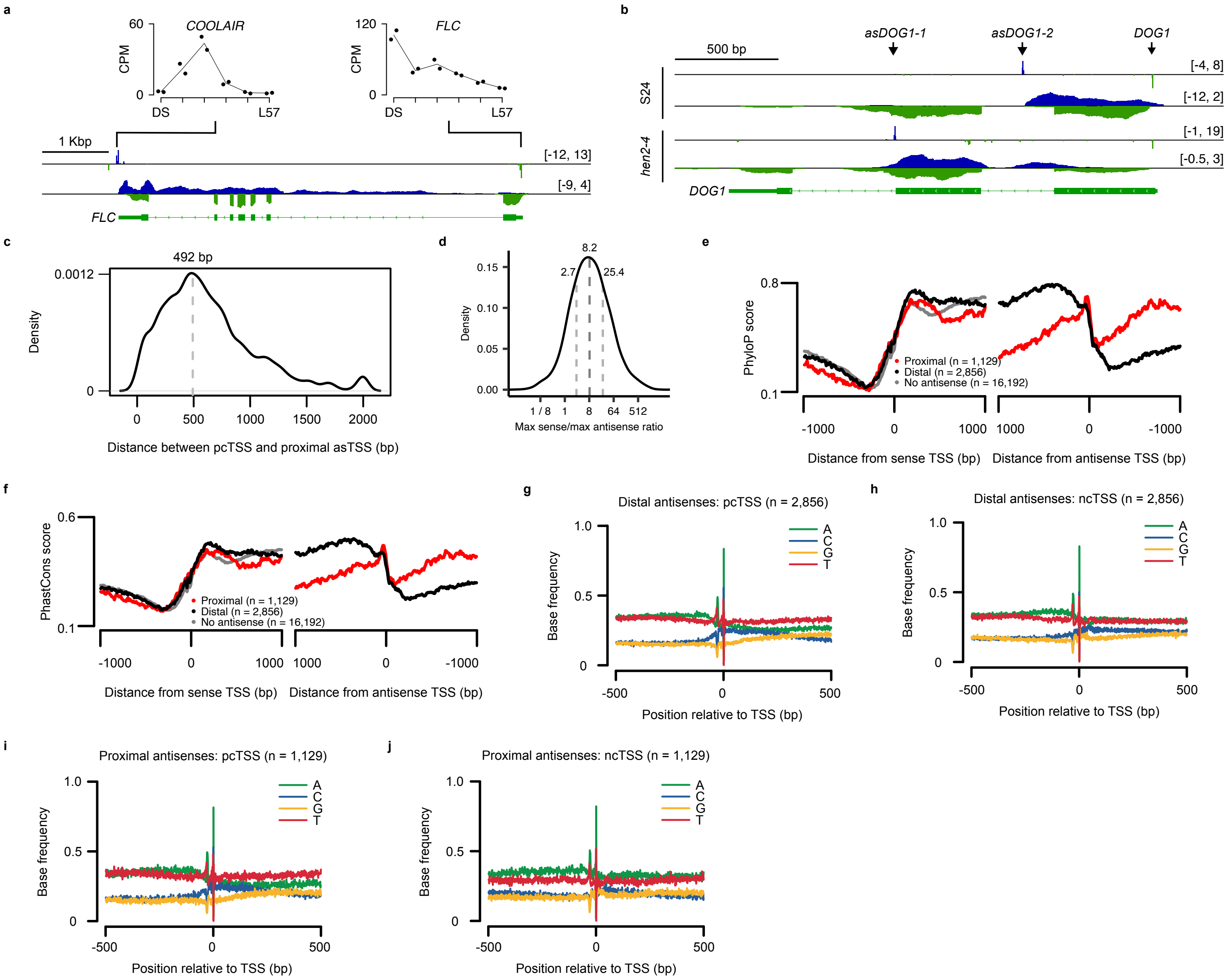

Supplementary Figure 5: Additional properties of antisense transcription.

(a) csRNA-seq and RNA-seq coverage tracks of the S72 time-point for the gene *FLC*, demonstrating the detection of the antisense transcript *COOLAIR* and its TSS (units in RPM). Also shown above are the csRNA-seq quantification data across all time-points for both *FLC* and *COOLAIR*.

(b) csRNA-seq and RNA-seq coverage tracks of the S24 and *hen2-4* samples for the gene *DOG1*, showing the two detected antisense TSSs active at different times during the seed-to-seedling transition. The antisense TSS *asDOG1-1* active in seedlings matches the location of the previously described *DOG1* antisense (Fedak et al., 2016). With the use of the csRNA-seq, we detect that a novel TSS, *asDOG1-2*, is active during stratification in a mutually exclusive fashion with *asDOG1-1*.

(c) Density plot showing the inter-TSS distances between protein coding TSSs and proximal antisense TSSs for genes with detected antisense transcription. The median distance is annotated using a dashed gray line.

(d) Density plot of the ratio of max csRNA-seq expression (on a $\log_{10}$ scale) between each pair of sense and antisense TSSs. The light gray dashed lines represent the IQR, and the darker gray dashed line the median.

(e) Average conservation of sense and antisense promoters for genes without a detected antisense as well as those with a proximal and distal antisense using PhyloP scores calculated from 63 plants (Tian et al., 2020). The coverage of scores is from 1 kb upstream and downstream of each primary TSS coordinate.

(f) Average conservation of sense and antisense promoters for genes without a detected antisense as well as those with a proximal and distal antisense using PhastCons scores calculated from 63 plants (Tian et al., 2020). The coverage of scores is from 1 kb upstream and downstream of each primary TSS coordinate.

(g) Average promoter base composition of protein coding TSSs for genes with distal antisense transcription in a 1 Kbp window centered around the TSS.

(h) Same as (g) for the promoters of the distal antisense TSSs.

(i) Same as (g) for the promoters of protein coding TSSs for genes with proximal antisense transcription.

(j) Same as (g) for the promoters of the proximal antisense TSSs.

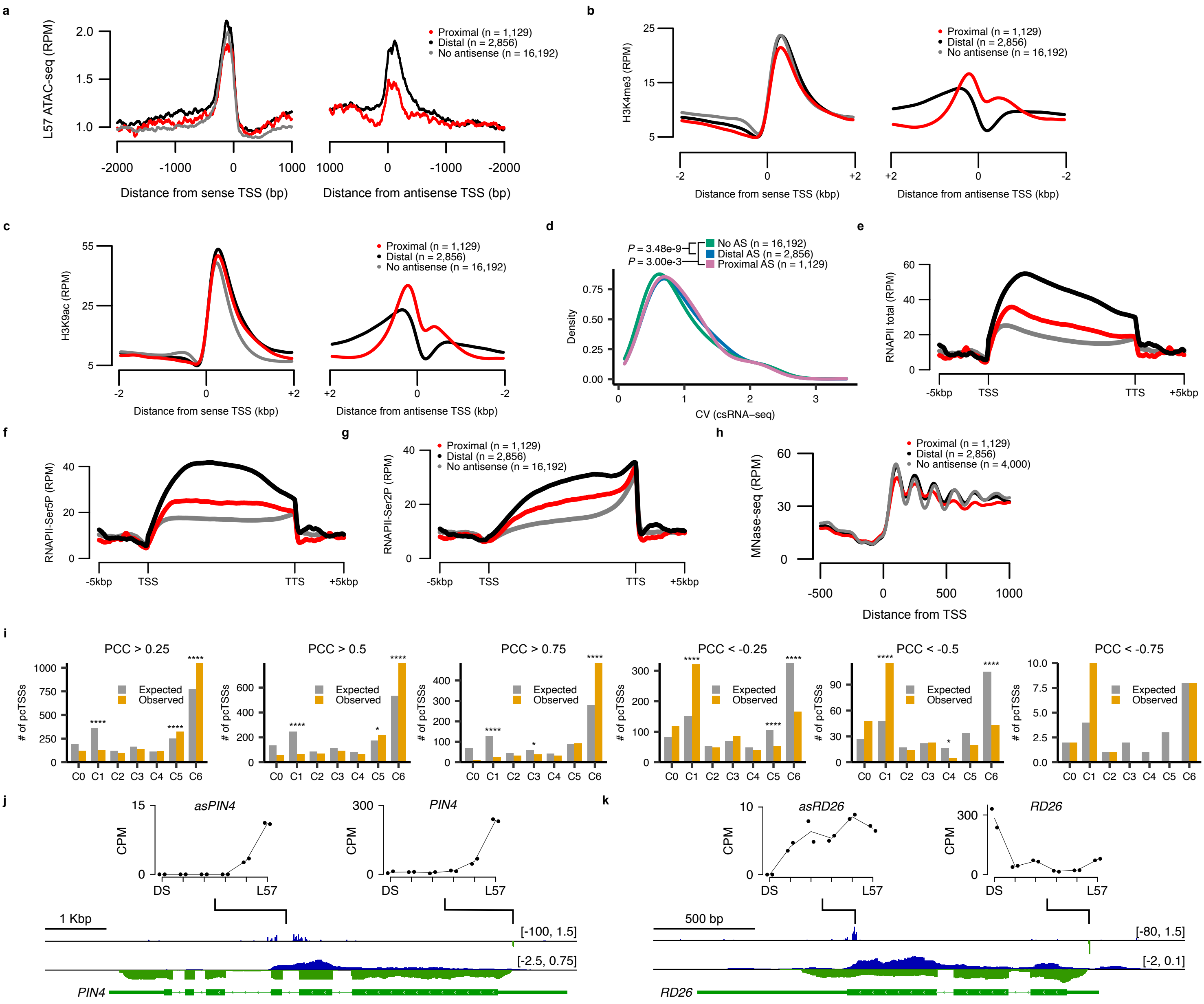

Supplementary Figure 6: Effects of antisense transcription on gene expression patterns.

(a) Average ATAC-seq read density at the promoters of protein coding TSSs for genes without antisense transcription, with distal antisense transcription, and with proximal antisense transcription. Also shown to the right are the equivalent data for the matching proximal and distal antisense promoters (from 2 Kbp upstream to 1 Kbp downstream).

(b) Same as (a) for average H3K4me3 ChIP-seq (Wollman et al., 2017) read density in a 4 Kbp region centered around the TSS.

(c) Same as (a) for average H3K9ac ChIP-seq (Chen et al., 2017) read density in a 4 Kbp region centered around the TSS.

(d) Density plot for the coefficient of variation of the csRNA-seq quantification data for protein coding TSSs with and without an antisense TSS (proximal or distal). Significance testing was performed using two-sided Mann-Whitney tests with Holm correction for multiple testing.

(e) Average total RNAPII ChIP-seq (Inagaki et al., 2021) over the gene bodies of genes with and without antisense transcription (proximal or distal), including 5 Kbp upstream and downstream regions.

(f) Same (e) for average RNAPII-Ser5P ChIP-seq (Inagaki et al., 2021).

(g) Same (e) for average RNAPII-Ser2P ChIP-seq (Inagaki et al., 2021).

(h) Average MNase-seq (Zhang et al., 2016) from 500 bp upstream to 1 Kbp downstream of TSSs with and without antisense transcription (proximal or distal).

(i) Expected versus observed number of protein coding TSSs of genes with antisense transcription in each csRNA-seq cluster (with C0 representing those without an assigned cluster) divided by various Pearson correlation coefficient thresholds. Significance testing was performed using Chi-squared tests without correcting for multiple testing (*P<0.05, **P<0.01, ***P<0.001, ****P<0.0001).

(j) csRNA-seq and RNA-seq coverage tracks of the *hen2-4* sample for the gene *PIN4*, demonstrating the detected positively-correlating *asPIN4* antisense (units in RPM). Also shown above are the matching csRNA-seq quantification data for all time-points of both sense and antisense TSSs.

(k) Same as (j) for the gene *RD26*, showing the detected negatively-correlating *asRD26* antisense.

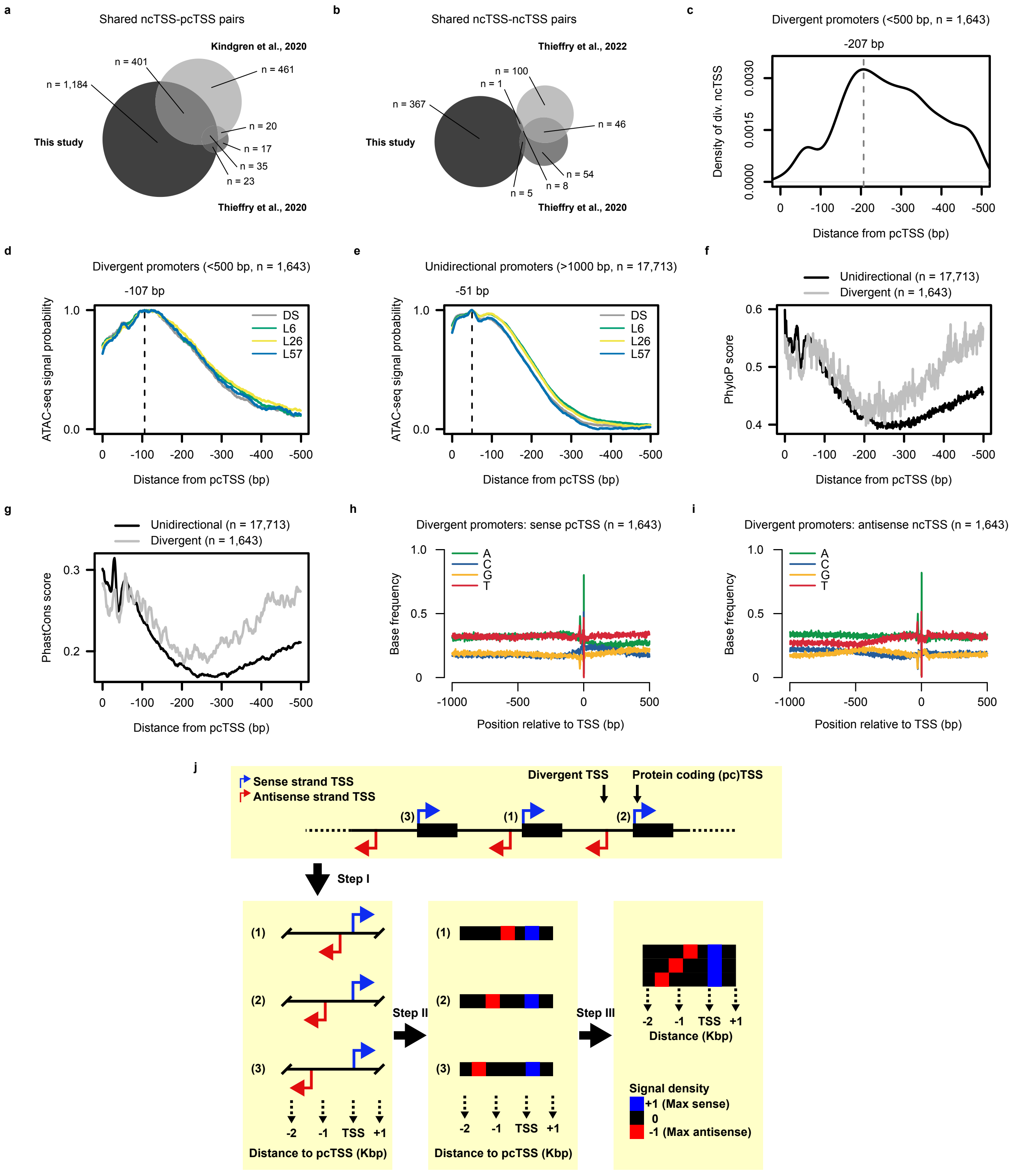

Supplementary Figure 7: Additional properties of divergent transcription.

(a) Venn diagram showing shared and unique bidirectional non-coding and protein coding TSS pairs (ncTSS-pcTSS; divergent promoters) detected in the csRNA-seq as well as those observed in previous studies (Kindgren et al., 2020; Thieffry et al., 2020).

(b) Venn diagram showing shared and unique bidirectional non-coding TSS pairs (ncTSS-ncTSS) detected in the csRNA-seq as well as those observed in previous studies (Thieffry et al., 2020, 2022).

(c) Density plot of the distance of the ncTSS from the pcTSS of divergent promoters detected in the csRNA-seq. The median distance is shown using a dashed gray line.

(d) Average normalized ATAC-seq read signal probability in the 500 bp upstream region from pcTSSs for divergent promoters. The median peak region of normalized ATAC-seq signal from all samples is shown using a dashed gray line.

(e) Same as (d) for unidirectional promoters (i.e. no detected divergent transcription).

(f) Average sequence conservation of divergent and unidirectional promoters using PhyloP scores calculated from 63 plants (Tian et al., 2020).

(g) Same as (f) using equivalent PhastCons scores (Tian et al., 2020).

(h) Average promoter base composition of protein coding TSSs for genes with divergent promoters, showing 1 Kbp upstream and 500 bp downstream of the TSS.

(i) Same as (h) from the perspective of the non-coding TSS in divergent promoters.

(j) Diagram explanation of the process of generating the heatmaps in Figure 6d. Divergent TSS regions across the genome are first collected in 3 Kbp chunks (2 Kbp upstream and 1 Kbp downstream of the pcTSS) and sorted in ascending order by the distance between the pcTSS and their divergent TSS (Step I). Then, the sense and antisense read densities are individually normalized between 0 to 1 and 0 to -1, respectively (Step II). Finally, all normalized signal vectors are assembled vertically and plotted in the style of a heatmap (Step III).

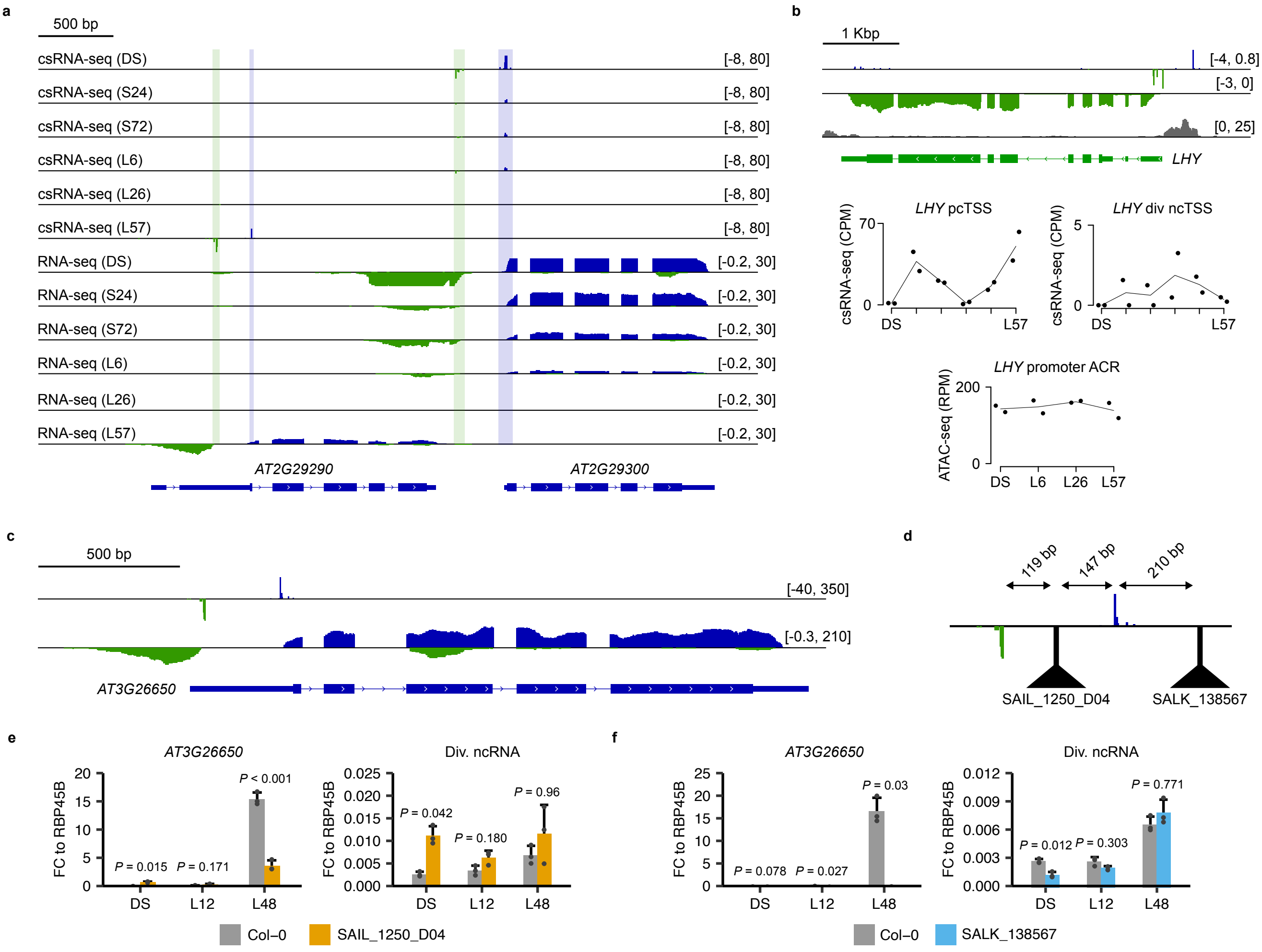

Supplementary Figure 8: Evidence of uncoordinated divergent transcription.

(a) csRNA-seq and RNA-seq read coverage tracks for two genes showing evidence of correlated divergent transcription across the seed-to-seedling transition (units in RPM). The TSSs are highlighted in green (antisense non-coding TSSs) and blue (sense protein coding TSSs).

(b) csRNA-seq, RNA-seq and ATAC-seq read coverage tracks of the L26 sample for the gene *LHY*, which shows evidence of non-correlating divergent transcription (units in RPM). The corresponding csRNA-seq quantification of the sense and antisense TSSs are shown below, also the ATAC-seq quantification of the *LHY* promoter ACR.

(c) csRNA-seq and RNA-seq read coverage tracks of the *hen2-4* sample for the gene *AT3G26650*, which shows evidence of divergent transcription and had available T-DNA insertion mutants interrupting the promoter region (units in RPM).

(d) Close-up of the csRNA-seq track from (I) showing the divergent promoter and the distances between the T-DNA insertion and the TSSs in the SAIL_1250_D04 and SALK_138567 mutant lines.

(e) RT-qPCR data of the *AT3G26650* mRNA and its divergent lncRNA in Col-0 and SAIL_1250_D04 plants. RNA was extracted for both genotypes from dry seeds (DS), 12 h seeds after moving to the light (L12), and 48 h seedlings after moving to the light (L48). Data are normalized to the constitutively expressed gene *RBP45B*. Significance testing was performed using two-sided Student's t-tests with Bonferroni correction for multiple testing. All experiments were performed with n = 3 biological replicates per time-point. Error bars show the standard deviation from the mean.

(f) Same as (e), comparing Col-0 and SALK_138567 plants.

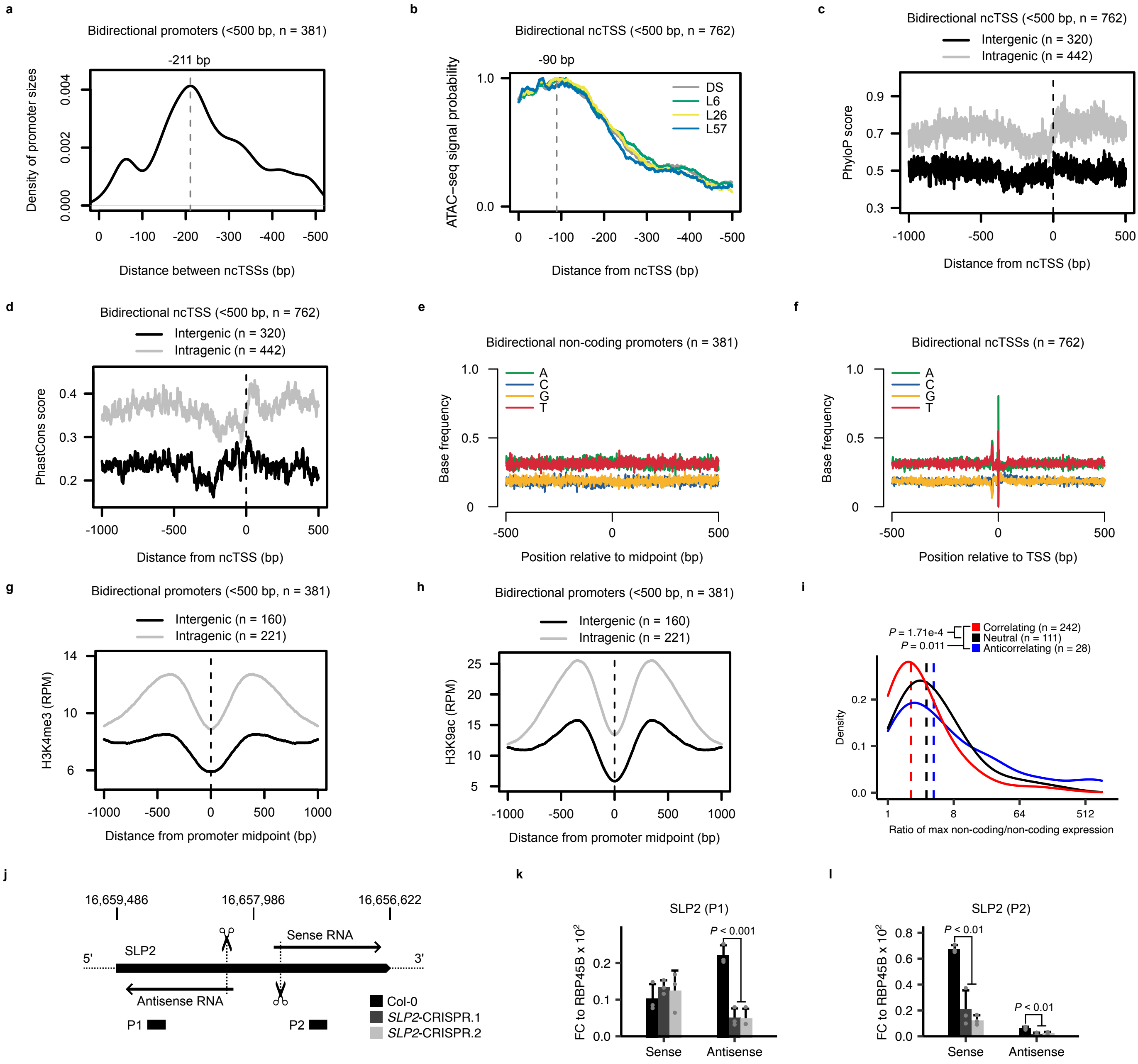

Supplementary Figure 9: Additional characteristics and validation of bidirectional non-coding promoters.

(a) Density plot of the inter-TSS distances between bidirectional non-coding TSS pairs. The median is shown as a dashed gray line.

(b) Average normalized ATAC-seq read signal probability in the 500 bp upstream region from non-coding TSSs for bidirectional non-coding promoters. The median peak region of normalized ATAC-seq signal from all samples is shown using a dashed gray line.

(c) Average sequence conservation of bidirectional non-coding promoters (from the perspective of the individual TSSs) using PhyloP scores calculated from 63 plants (Tian et al., 2020).

(d) Same as (c) using equivalent PhastCons scores (Tian et al., 2020).

(e) Average promoter base composition of bidirectional non-coding promoters, showing a 1 Kbp region centered at the midpoint between the two TSSs.

(f) Same as (e) from the perspective of the individual non-coding TSSs from bidirectional non-coding promoters.

(g) Average H3K4me3 ChIP-seq (Wollman et al., 2017) read density over bidirectional non-coding promoters, in a 2 Kbp region centered at the midpoint between the two TSSs. The data is shown separately for intragenic and intergenic bidirectional non-coding promoters.

(h) Same as (g) using H3K9ac ChIP-seq (Chen et al., 2017) data.

(i) Density plot of the ratio (on a $\log_2$ scale) of csRNA-seq signal between each TSS pair in bidirectional non-coding promoters, grouped by their Pearson correlation coefficient (anti-correlating: less than -0.25; correlating: greater than 0.25). Dashed lines represent the median ratio within each group. Significance testing between correlation groups was performed using two-sided Mann-Whitney tests with Holm correction for multiple testing.

(j) Schematic of the *SLP2* gene, which has an intragenic bidirectional non-coding promoter expressed in dry seeds and early stratification. The positions of the bidirectional non-coding transcripts, primer pairs used to test their abundance (P1 and P2), and location of the gRNA target sites used to generate the CRISPR-Cas9 deletion mutants (represented as scissors) are shown.

(k) RT-qPCR data of the P1 primer pair using cDNA generated from the sense and antisense strands in Col-0 and the two CRISPR-Cas9 mutant lines, normalized to *RBP45B* using RNA extracted from dry seeds. In this case as there is no transcription over the sense strand in the dry seed, deleting the bidirectional non-coding promoter does not result in any difference between Col-0 and the deletion mutants. Instead a significant drop can be detected using antisense-specific cDNA, which shows higher abundance compared to the sense strand. Statistical testing for (k) and (l) were performed using one-way ANOVA with Tukey's Honest Significant Difference post hoc tests. All experiments were performed with n = 3 biological replicates per time-point. Error bars show the standard deviation from the mean.

(l) Same as (k) for the P2 primer pair. Increased abundance of transcription is detected on the sense strand as compared to the antisense strand, which is strongly reduced as a result of the deletion of the bidirectional non-coding promoter.

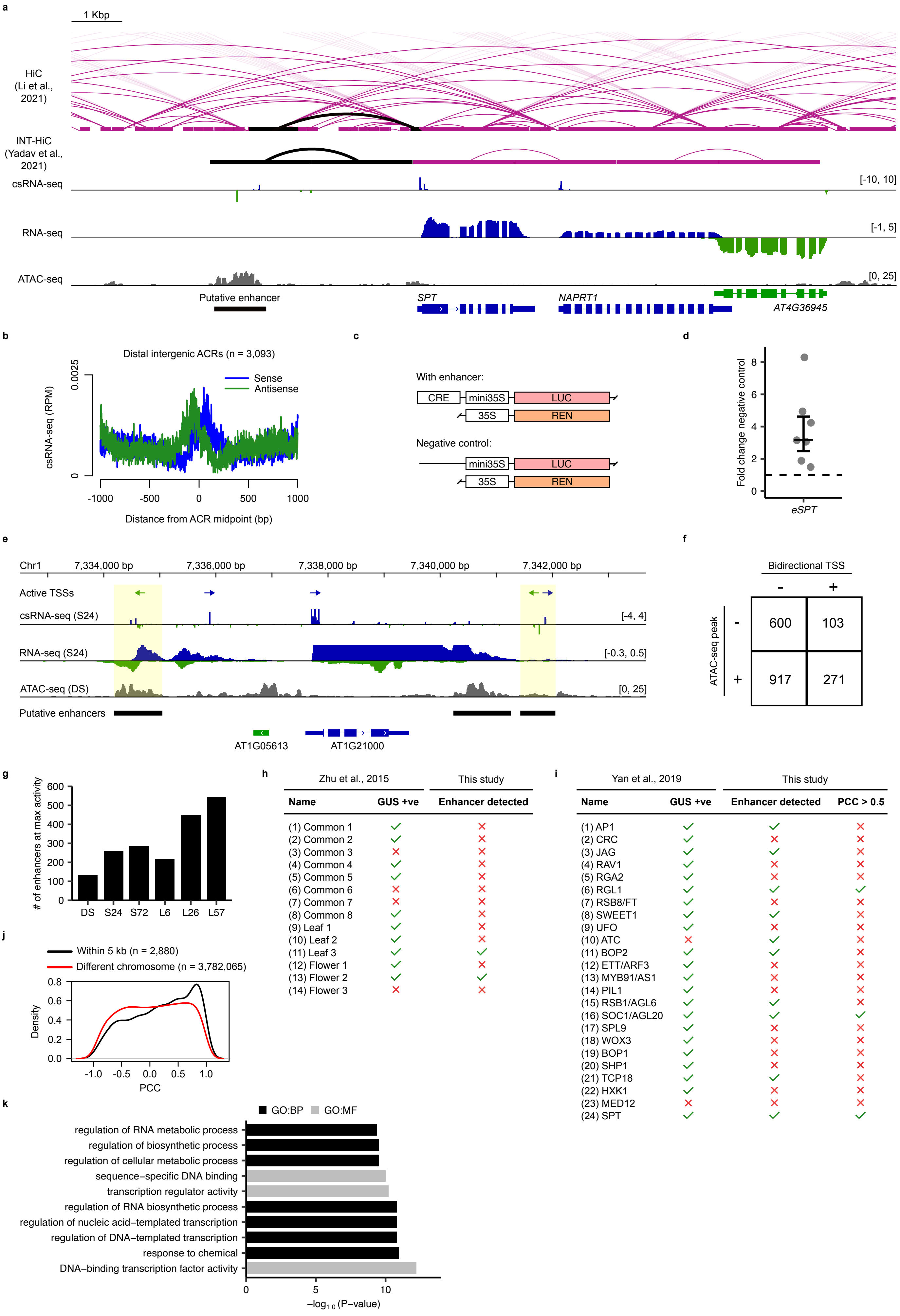

Supplementary Figure 10: Identification of transcriptionally active enhancers.

(a) csRNA-seq, RNA-seq and ATAC-seq read coverage tracks of the L26 sample showing the genomic region containing the *SPT* gene and a putative upstream intergenic enhancer (*eSPT*) with bidirectional non-coding transcriptional activity (units in RPM). Also shown above are chromatin interaction data from previously published HiC (Li et al., 2021) and INT-HiC (Yadav et al., 2021) datasets. Loops which overlap the putative enhancer and the *SPT* promoter region are bolded and drawn in black.

(b) Average csRNA-seq signal (from all Col-0 samples merged together) at distal intergenic ACRs (at least 1 Kbp away from a TSS) where no TSS peaks were detected.

(c) Overview of the pGreen II 0800 mini35S:LUC reporter constructs used to test the ability of putative enhancers to activate transcription. The putative enhancers are directly upstream of a minimal 35S promoter, which is unable to express the *LUC* gene without an enhancer element. A construct without any upstream enhancer sequence is used as the negative control. The Renilla *LUC* (*REN*) gene expressed by a 35S promoter is used as an internal control.

(d) Enhancer assay using the putative enhancer found in the upstream intergenic ACR of the *SPT* gene using the system described in (c). Individual N. benthamiana leaves were infiltrated with the *SPT*-containing construct as well as the negative control construct. The *LUC* expression for each replicate was normalized to its *REN* expression, then calculated as a fold-change to the normalized *LUC* expression of the negative control from the same leaf (n = 7). A dashed line represents a fold-change of 1 (i.e., no increased expression of *LUC* compared to the negative control). The lower, middle and upper hinges correspond to first quartile, median, and third quartile, respectively. The lower and upper whiskers extend to the minimal/maximal value respectively or 1.5 times the interquartile range, whichever is closer to the median.

(e) csRNA-seq, RNA-seq and ATAC-seq read coverage tracks of the S24 and DS samples showing putative enhancers in intergenic regions upstream and downstream of the gene *AT1G21000* (units in RPM). This example demonstrates putative enhancers containing unidirectional and bidirectional non-coding transcription (based on the detection of TSS peaks in the csRNA-seq).

(f) Number of putative enhancers with detectable ACR peaks and bidirectional non-coding TSS peaks.

(g) The number of putative enhancers with maximum csRNA-seq expression per time-point.

(h) The list of tested candidate enhancer regions from Zhu et al. (2015). This study tested their ability to enhance transcription of a *GUS* gene with a minimal 35S promoter. Candidate enhancers with detectable *GUS* expression have a green checkmark in the “GUS +ve” column. If the candidate enhancer regions overlap a putative enhancer detected in this study, they are marked with a green checkmark in the “Enhancer detected” column.

(i) Same as (i) for candidate enhancer regions from Yan et al. (2019). This study assigned putative gene targets of the enhancers. For those with a detected putative enhancer in this study, we calculated the Pearson correlation coefficient (PCC) between the enhancer activity and the gene csRNA-seq expression. Those with a positive correlation above 0.5 are marked with a green checkmark.

(j) Density plot of the Pearson correlation coefficients (PCC) between enhancer activities of putative enhancers found in this study and protein coding TSSs within 5 Kbp. This is compared with PCCs between putative enhancers and the same protein coding TSSs but from different chromosomes as a way to see the random distribution of possible PCCs.

(k) Top 10 enriched gene ontology terms of genes whose csRNA-seq expression correlated highly (Pearson correlation coefficient greater than 0.5) with a nearby putative enhancer (less than 5 Kbp between the protein coding TSS and the putative enhancer).

Supplementary Table 1: Primer sequences used in this study.

This table contains all primers used for genotyping mutant lines, cloning, smFISH and qPCR experiments (see methods).

Name	Description	Sequence
hen2-4F	Genotyping primer for the hen2-4 mutant.	CAGAAACCGTAAATGTTTTGGAAT
hen2-4R	Genotyping primer for the hen2-4 mutant.	ATTGTCCTTCGGCACGACTC
rrp4-2F	Genotyping primer for the rrp4-2 mutant.	CTATTCCCGTCAACCATGACG
rrp4-2R	Genotyping primer for the rrp4-2 mutant.	CATCGACCTCGGAAGTTCCAGGT
LBb1.3	Genotyping SALK T-DNA insertion lines.	ATTTTGCCGATTTCGGAAC
LBb1	Genotyping SAIL T-DNA insertion lines.	GCCTTTTCAGAAATGGATAAATAGCCTTGCTTCC
RBP45B-F	RT-qPCR control optimal for germination.	GCATGTGAAAATACCCGCTG
RBP45B-R	RT-qPCR control optimal for germination.	TTCCTCTGCACAGCTCTTCTC
M13-F	Genotyping/sequencing primer for the enhancer insertion region of the pGreen II 0800 mini35S:LUC plasmid.	GTAAAACGACGGCCAGT
5pLUC-R	Genotyping/sequencing primer for the enhancer insertion region of the pGreen II 0800 mini35S:LUC plasmid.	CCGGGCCTTTCTTTATGTTTT
mini35S_frag_s	The sense mini35S promoter sequence with a five prime partial BamHI site.	GATCcgcaagacccttcctctatataaggaagttcatttcatttggagaggac
mini35S_frag_as	The antisense mini35S promoter sequence with a five prime partial NotI site.	GGCCgtcctctccaaatgaaatgaacttccttatatagaggaagggtcttgcg
SPT_enh-F	To amplify the region containing the putative intergenic enhancer upstream of SPT, with a five prime KpnI site.	ATAGGTACCAGCGAAGTCTCAGCTTTTTCG
SPT_enh-R	To amplify the region containing the putative intergenic enhancer upstream of SPT, with a five prime SalI site.	ATAGTCGACTGAAACCGATATCACCATGCCA
SAIL_1250_D04-F	Genotyping/sequencing primer for the SAIL_1250_D04 T-DNA insertion line.	GCAGCTTCAGTGGTCATCTTC
SAIL_1250_D04-R	Genotyping/sequencing primer for the SAIL_1250_D04 T-DNA insertion line.	GAAGGTACCTTGGGGACAGAG
SALK_138567-F	Genotyping/sequencing primer for the SALK_138567 T-DNA insertion line.	GTCAGACAAATGGAGAGCAGC
SALK_138567-R	Genotyping/sequencing primer for the SALK_138567 T-DNA insertion line.	TGATAACCTTCTTGGCACCAG
SALK_073206-F	Genotyping/sequencing primer for the SALK_073206 T-DNA insertion line.	TATTAATAGTGGCCTGGCGAG
SALK_073206-R	Genotyping/sequencing primer for the SALK_073206 T-DNA insertion line.	AAACTTGCCCCAACTCTTTTC
SALK_201027-F	Genotyping/sequencing primer for the SALK_201027 T-DNA insertion line.	TGTGGGGTTGTTTCTTTCTTG
SALK_201027-R	Genotyping/sequencing primer for the SALK_201027 T-DNA insertion line.	CTGCCCTAATCAAGTCGACAC
AT1G04170_div-F	RT-qPCR primer to test the abundance of the divergent non-coding transcript for the AT1G04170 gene.	AGCGTTCGTGTTCCTCTATCT
AT1G04170_div-R	RT-qPCR primer to test the abundance of the divergent non-coding transcript for the AT1G04170 gene.	CGTTTCTGACTTCAACAGCCG
AT1G04170-F	RT-qPCR primer to test the abundance of the AT1G04170 mRNA.	ACTGACTGCGCCTGTTTGTA
AT1G04170-R	RT-qPCR primer to test the abundance of the AT1G04170 mRNA.	GCCAATGCTTTTCCACACGT
AT3G26650_div-F	RT-qPCR primer to test the abundance of the divergent non-coding transcript for the AT3G26650 gene.	AGAGCAGAGGTTCTTAGGACT
AT3G26650_div-R	RT-qPCR primer to test the abundance of the divergent non-coding transcript for the AT3G26650 gene.	AGGCATACAAGGTCTGTGGT
AT3G26650-F	RT-qPCR primer to test the abundance of the AT3G26650 mRNA.	ATCGCTCTCCGTGTACCAAC
AT3G26650-R	RT-qPCR primer to test the abundance of the AT3G26650 mRNA.	AAGCAGCGTTGACTTCCTCA
SLP2_P1-F	RT-qPCR primer to test the abundance of the putative antisense bidirectional non-coding transcript.	GATGTCCGTCGCCGAGAATA
SLP2_P1-R	RT-qPCR primer to test the abundance of the putative antisense bidirectional non-coding transcript.	CTTCGTCTCTTCCTCTGCCG
SLP2_P2-F	RT-qPCR primer to test the abundance of the putative sense bidirectional non-coding transcript.	ACCTACAGGTTTGCCGTCAG
SLP2_P2-R	create sense strand-specific cDNA.	GCACCACTAACGTGAGGACA
SLP2_P1-R2	Used to create antisense strand-specific cDNA.	TGGTATAGCACCGAGTTCGC
SLP2-guide4-BsF	SLP2 CRISPR cloning.	ATATATGGTCTCGATTGTCTCCGGTAGAGGACCAAAGTT
SLP2-guide4-F0	SLP2 CRISPR cloning.	TGTCTCCGGTAGAGGACCAAAGTTTTAGAGCTAGAAATAGC
SLP2-guide3-R0	SLP2 CRISPR cloning.	AACGTGGTTTATCCCGGTAAATCAATCTCTTAGTCGACTCTAC
SLP2-guide3-BsR	SLP2 CRISPR cloning.	ATTATTGGTCTCGAAACGTGGTTTATCCCGGTAAATCAA
#5_sense_unspl_1	Probes for smFISH.	aaccttttttttttttccct
#5_sense_unspl_2	Probes for smFISH.	caagtccctaaagggttttt
#5_sense_unspl_3	Probes for smFISH.	cgcagcttgaagaagatgca
#5_sense_unspl_4	Probes for smFISH.	aaacgaacctgagcgatgcg
#5_sense_unspl_5	Probes for smFISH.	ttacgattagattccccgaa
#5_sense_unspl_6	Probes for smFISH.	cagattctatacctgagctc
#5_sense_unspl_7	Probes for smFISH.	ctacatctgccctaatcaag
#5_sense_unspl_8	Probes for smFISH.	aaccctaaccctagttaatc
#5_sense_unspl_9	Probes for smFISH.	cttctgctcctgcttaaaac
#5_sense_unspl_1	Probes for smFISH.	ttttaagatcttgctcagcc
#5_sense_unspl_1	Probes for smFISH.	gatgtagcacagttacatcc
#5_sense_unspl_1	Probes for smFISH.	gcgagaaatgacctcaggag
#5_sense_unspl_1	Probes for smFISH.	gccagtttcttcaacatcaa
#5_sense_unspl_1	Probes for smFISH.	catgaccaatggttcctgaa
#5_sense_unspl_1	Probes for smFISH.	caacagtggactttccatga
#5_sense_unspl_1	Probes for smFISH.	gcctctaggattacaattgt
#5_sense_unspl_1	Probes for smFISH.	agaaatcacttcacccactc
#5_sense_unspl_1	Probes for smFISH.	acggacagtctgtacagaga
#5_sense_unspl_1	Probes for smFISH.	gcatatccaagcttaatggt
#5_sense_unspl_2	Probes for smFISH.	ttctcatcctcacatttgta
#5_sense_unspl_2	Probes for smFISH.	actcacttgtagcacattgg
#5_sense_unspl_2	Probes for smFISH.	agttgttagaccttgactca
#5_sense_unspl_2	Probes for smFISH.	gtaggccctaatacaacaga
#5_sense_unspl_2	Probes for smFISH.	aatccggggacatcacaatt
#5_sense_unspl_2	Probes for smFISH.	accgggcaatcaacgaatga
#5_sense_unspl_2	Probes for smFISH.	aaatcatgcaggtggagcac
#5_sense_unspl_2	Probes for smFISH.	catcaaccctcactctttaa
#5_sense_unspl_2	Probes for smFISH.	catgagaatatcgtgaccct
#5_sense_unspl_2	Probes for smFISH.	aaagtagtgcaccatccatg
#5_sense_unspl_3	Probes for smFISH.	ggacaagtttcatttgcagc
#5_sense_unspl_3	Probes for smFISH.	atgttcagacgtttgtggtt
#5_sense_unspl_3	Probes for smFISH.	gttgcataatctcaacggca
#5_sense_unspl_3	Probes for smFISH.	aattgcctcgtgctgattaa
#5_sense_unspl_3	Probes for smFISH.	cagtgttctgcaacaggatg
#5_sense_unspl_3	Probes for smFISH.	ggggtgacacaaaattcctc
#5_sense_unspl_3	Probes for smFISH.	atcaacctcataaccaggtt
#5_sense_unspl_3	Probes for smFISH.	aacgataccaggtcggattt
#5_sense_unspl_3	Probes for smFISH.	tgttccgcgtagagtgaaat
#5_sense_unspl_3	Probes for smFISH.	ctccaggaacagcaaactga
#5_sense_unspl_4	Probes for smFISH.	tgttgttccaactcctatta
#5_sense_unspl_4	Probes for smFISH.	atctgcacgagtgagagttg
#5_sense_unspl_4	Probes for smFISH.	gaaccgatttcaccaaggac
#5_sense_unspl_4	Probes for smFISH.	gagtatggttgtgaggagtt
#5_sense_unspl_4	Probes for smFISH.	tgttctcactcccaacaaac
#5_sense_unspl_4	Probes for smFISH.	cagaatctctcctttcgtta
#5_sense_unspl_4	Probes for smFISH.	gtttagccagatcgacttta
#5_sense_unspl_4	Probes for smFISH.	tttgctggtacaaacaggcg
#5_sense_unspl_4	Probes for smFISH.	cccccaacgagaaaaagagt
Actin7_-995F	ChIP-qPCR primers to test RNAPII accumulation in seeds.	tgggtctcatatagaacactcacaaaggt
Actin7_-832R	ChIP-qPCR primers to test RNAPII accumulation in seeds.	gaccaaaacccgaataggagcaaga
Actin7_55F	ChIP-qPCR primers to test RNAPII accumulation in seeds.	cgtttcgctttccttagtgttagct
Actin7_188R	ChIP-qPCR primers to test RNAPII accumulation in seeds.	agcgaacggatctagagactcaccttg
Actin7_886F	ChIP-qPCR primers to test RNAPII accumulation in seeds.	tgccccgagagcagtgttcc
Actin7_992R	ChIP-qPCR primers to test RNAPII accumulation in seeds.	tggactgagcttcatcaccaacg
Actin7_2477F	ChIP-qPCR primers to test RNAPII accumulation in seeds.	gtatcgggtgacaatgcagctattatgt
Actin7_2561R	ChIP-qPCR primers to test RNAPII accumulation in seeds.	tgctggagtaaaacataagccactcac
IGN5-set1-F	ChIP-qPCR primers to test RNAPII accumulation in seeds.	gacatggttgggtccttgtt
IGN5-set1-R	ChIP-qPCR primers to test RNAPII accumulation in seeds.	aatgtcggccaatcttcttg
DOG1-ChIP-P3-F	ChIP-qPCR primers to test RNAPII accumulation in seeds.	GGCTCTCAAAGGTTTCCTTG
DOG1-ChIP-P3-R	ChIP-qPCR primers to test RNAPII accumulation in seeds.	GCATCAAATAGGAAGCGACAG
DOG1-ChIP-C1-F	ChIP-qPCR primers to test RNAPII accumulation in seeds.	CTGATCTTGCTCACCGATGTAG
DOG1-ChIP-C1-R	ChIP-qPCR primers to test RNAPII accumulation in seeds.	ACGGATCTCAGTTTGTGACC
DOG1-ChIP-C2-F	ChIP-qPCR primers to test RNAPII accumulation in seeds.	ATATTCCCATCGCCACTGTG
DOG1-ChIP-C2-R	ChIP-qPCR primers to test RNAPII accumulation in seeds.	TTGTCGAGAGCTTGATCCAC
DOG1-ChIP-C3-F	ChIP-qPCR primers to test RNAPII accumulation in seeds.	ATTCACGTCGTGGCATTTTGCG
DOG1-ChIP-C3-R	ChIP-qPCR primers to test RNAPII accumulation in seeds.	CTCTCATTATTTGTCGTCTCCGTGG

Supplementary Table 2: Links to external datasets used in this study.

External NGS datasets and Arabidopsis thaliana conservation scores were downloaded in raw (FASTQ) or processed (BigWig, BEDPE) format depending on availability (see methods).

Name	Description	Data type	Source/Accession ID	Citation
MNase-seq	Leaf MNase-seq nucleosomal reads.	BigWig	https://bioinfor.yzu.edu.cn/download/plantdhs/Ath_leaf_NPS.bw	T. Zhang et al., 2016
RNAPII	Seedling total RNAPII ChIP-seq.	FASTQ	DRA010413	Inagaki et al., 2021
RNAPII-Ser2P	Seedling RNAPII-Ser2P ChIP-seq.	FASTQ	DRA010413	Inagaki et al., 2021
RNAPII-Ser5P	Seedling RNAPII-Ser5P ChIP-seq.	FASTQ	DRA010413	Inagaki et al., 2021
H3K4me3	Seedling H3K4me3 ChIP-seq.	FASTQ	GSE96834	Wollmann et al., 2017
H3K9ac	Seedling H3K9ac ChIP-seq.	FASTQ	GSE79524	C. Chen et al., 2017
H3K4me1	Seedling H3K4me1 ChIP-seq.	FASTQ	DRA010413	Inagaki et al., 2021
H3K36me3	Seedling H3K36me3 ChIP-seq.	FASTQ	GSE96834	Wollmann et al., 2017
H2AZ	Seedling H2AZ ChIP-seq.	FASTQ	GSE96834	Wollmann et al., 2017
H2AK121ub	Seedling H2AK121ub ChIP-seq.	FASTQ	GSE89357	Y. Zhou et al., 2017
H3K27me3	Seedling H3K27me3 ChIP-seq.	FASTQ	GSE89357	Y. Zhou et al., 2017
GRO-cap	Seedling GRO-cap.	FASTQ	GSE83108	Hetzel et al., 2016
HiC	Seedling HiC.	BEDPE	https://static-content.springer.com/esm/art%3A10.1038%2Fs41477-021-01004-x/MediaObjects/41477_2021_1004_MOESM6_ESM.xlsx	L. Li et al., 2021
INT-HiC	Endosperm INT-HiC.	BEDPE	https://oup.silverchair-cdn.com/oup/backfile/Content_public/Journal/nar/49/8/10.1093_nar_gkab191/1/gkab191_supplemental_files.zip?Expires=1688479597&Signature=x8HGjtgNXfv6alCNCqWDEzqNPAPr-jwTI7Ka7ncSay~J~Jf1nQ9947Jr0ikXsn4LAX0vsgsAS2ZOoFeF~DKAyI1VI3PPRIVqcQwZtXJkeTlER3IiDrnBJDb0ustA6SQN8IQL1~vjnClbzVXhVOJwJj0vtrDi3xNxAaHpWhD2Hk-3yWsjrpBpOgZGsemJEoQCDXZker1SD0-Ubopu34neeHaAn2o07CpW2uko0MHmPCeE0cg9wtCVziWJpn0qG--TVCmY2DXO2EJ~LPOf~CQpYvU1TJan7TufamVs98eT-jbkaPqNh1FhgRPzBQN5mfgpM49sQHUTy1Mwf1NSLz8hNA__&Key-Pair-Id=APKAIE5G5CRDK6RD3PGA	Yadav et al., 2021
CAGE	Seedling CAGE.	BigWig	https://www.ncbi.nlm.nih.gov/geo/download/?acc=GSE136356&format=file	Thieffry et al., 2020
PhyloP	Arabidopsis thaliana PhyloP conservation scores from 63 plants.	BigWig	http://plantregmap.gao-lab.org/download.php#comparative-genomics	Tian et al., 2020
PhastCons	Arabidopsis thaliana PhastCons conservation scores from 63 plants.	BigWig	http://plantregmap.gao-lab.org/download.php#comparative-genomics	Tian et al., 2020
ABI5 DAP-seq	ABI5 DAP-seq from ABA-treated seedlings.	BigWig	http://systemsbiology.cau.edu.cn/chromstates/At_bwfile/ABI5-SRX670509.bw	Y. Liu et al., 2018; O'Malley et al., 2016

Supplementary Table 3: Raw and filtered read counts for NGS data generated in this study.

The number of raw (total), mapped and filtered reads for each csRNA-seq, sRNA-seq, RNA-seq and ATAC-seq samples are provided.

Sample	Replicate	Experiment	Sequencing strategy	Total reads	Mapped reads	Filtered reads
DS	1	csRNA-seq	SE75	19139175	9106121	2437456
DS	2	csRNA-seq	SE75	16922100	10162944	3201888
S24	1	csRNA-seq	SE75	21940651	13861705	3324713
S24	2	csRNA-seq	SE75	28410400	16039220	3164850
S72	1	csRNA-seq	SE75	27941550	17436844	3247968
S72	2	csRNA-seq	SE75	23032593	14753183	3573860
L6	1	csRNA-seq	SE75	30254912	18965099	7313720
L6	2	csRNA-seq	SE75	17818146	12019732	4737713
L26	1	csRNA-seq	SE75	21858813	17194998	7446142
L26	2	csRNA-seq	SE75	23912077	19241156	7812405
L57	1	csRNA-seq	SE75	18882014	12662875	4550291
L57	2	csRNA-seq	SE75	21586808	14001012	5128857
hen2-4	1	csRNA-seq	SE75	18779213	15260001	5488276
hen2-4	2	csRNA-seq	SE75	18341497	13706983	5125184
rrp4-2	1	csRNA-seq	SE75	17915348	14761330	5822523
rrp4-2	2	csRNA-seq	SE75	19854173	11968603	4031775
DS	1	sRNA-seq	SE75	24099290	21492994	3207751
DS	2	sRNA-seq	SE75	21696803	19320189	2830985
S24	1	sRNA-seq	SE75	20721551	17008788	2632560
S24	2	sRNA-seq	SE75	20626446	15684888	2484544
S72	1	sRNA-seq	SE75	26887389	20353573	2140601
S72	2	sRNA-seq	SE75	20993743	14880805	1494974
L6	1	sRNA-seq	SE75	22830763	18616013	2325167
L6	2	sRNA-seq	SE75	22462768	19119814	2414518
L26	1	sRNA-seq	SE75	25271034	21898821	3281555
L26	2	sRNA-seq	SE75	24188194	22286045	3117980
L57	1	sRNA-seq	SE75	21851747	20310113	2724054
L57	2	sRNA-seq	SE75	25303166	23035741	3439387
hen2-4	1	sRNA-seq	SE75	23261814	20197431	2935019
hen2-4	2	sRNA-seq	SE75	23307277	20127440	3178108
rrp4-2	1	sRNA-seq	SE75	24770556	21822790	3474350
rrp4-2	2	sRNA-seq	SE75	16234577	13314446	2027525
DS	1	RNA-seq	PE125	137179388	131735040	131735040
DS	2	RNA-seq	PE125	163244132	158219131	158219131
S24	1	RNA-seq	PE125	371897030	358912184	358912184
S24	2	RNA-seq	PE125	404175970	390230892	390230892
S72	1	RNA-seq	PE125	367558592	353696972	353696972
S72	2	RNA-seq	PE125	398327260	380612413	380612413
L6	1	RNA-seq	PE125	162487494	157502455	157502455
L6	2	RNA-seq	PE125	157017428	151522878	151522878
L26	1	RNA-seq	PE125	161084294	155291808	155291808
L26	2	RNA-seq	PE125	172055942	165408559	165408559
L57	1	RNA-seq	PE125	151048816	145755348	145755348
L57	2	RNA-seq	PE125	180904132	173271622	173271622
hen2-4	1	RNA-seq	PE125	418904674	403470287	403470287
hen2-4	2	RNA-seq	PE125	429883650	411060307	411060307
rrp4-2	1	RNA-seq	PE125	400326536	386283530	386283530
rrp4-2	2	RNA-seq	PE125	444716774	427763505	427763505
DS	1	ATAC-seq	PE50	110076820	105219887	10911980
DS	2	ATAC-seq	PE50	110988952	108461758	11053799
L6	1	ATAC-seq	PE50	92377268	91837412	7666285
L6	2	ATAC-seq	PE50	87427980	87005450	8367244
L26	1	ATAC-seq	PE50	81720090	81427988	5560114
L26	2	ATAC-seq	PE50	119163326	118827453	7233412
L57	1	ATAC-seq	PE50	89169864	88452716	5138295
L57	2	ATAC-seq	PE50	100244846	99602417	5995526

Supplementary Note 1

The selected time-points accurately capture key developmental stages of germination

Our aim was to capture transcription initiation events associated with major developmental checkpoints during the seed-to-seedling transition, including early germination, the transition between germinative and post-germinative growth, and the start of the vegetative stage. To validate this we examined chloroplast read content of the RNA-seq libraries as a proxy of seedling development. Using this approach we observed similar basal levels of chloroplast-originating transcription during early germination (from DS to L6), until an increase could be detecting starting from our sample representing the transition to post-germinative growth (L26), which ultimately culminated in nearly half of all transcription captured by the RNA-seq originated from chloroplastic RNA in our seedling sample (L57; Supplementary Figure 11). This approach thus confirmed we had captured all relevant stages of the seed-to-seedling transition.

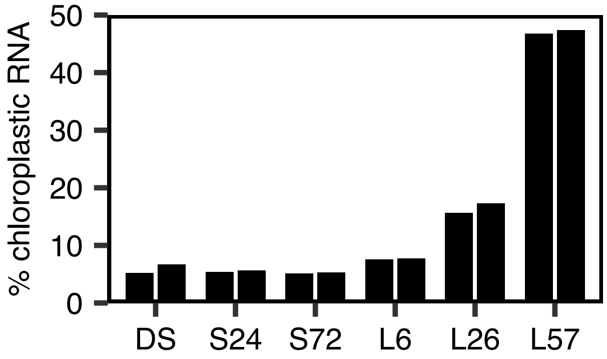

Supplementary Figure 11: Total chloroplastic RNA detected in the RNA-seq over time.
The percent of reads in all RNA-seq reads mapping to the chloroplast.

The csRNA-seq enriches for capped-small RNAs and depletes other small RNAs

As the range of sizes of capped-small RNAs captured by the csRNA-seq (20-70 nt) include those of small RNAs in Arabidopsis (21-24 nt) (Mallory & Vaucheret, 2006), we compared the abundances of read sizes from our sRNA-seq and csRNA-seq samples. Using this approach, we could note an accumulation of sRNAs of sizes 21-24 nt in all of the sRNA-seq libraries, which were clearly depleted in all csRNA-seq libraries (Supplementary Figure 12). Additionally we could note the accumulation of various small RNA species in the 30-40 nt range in some sRNA-seq libraries which were also depleted in the csRNA-seq libraries. These results suggest successful enrichment of capped-small RNAs and depletion of uncapped-small RNAs.

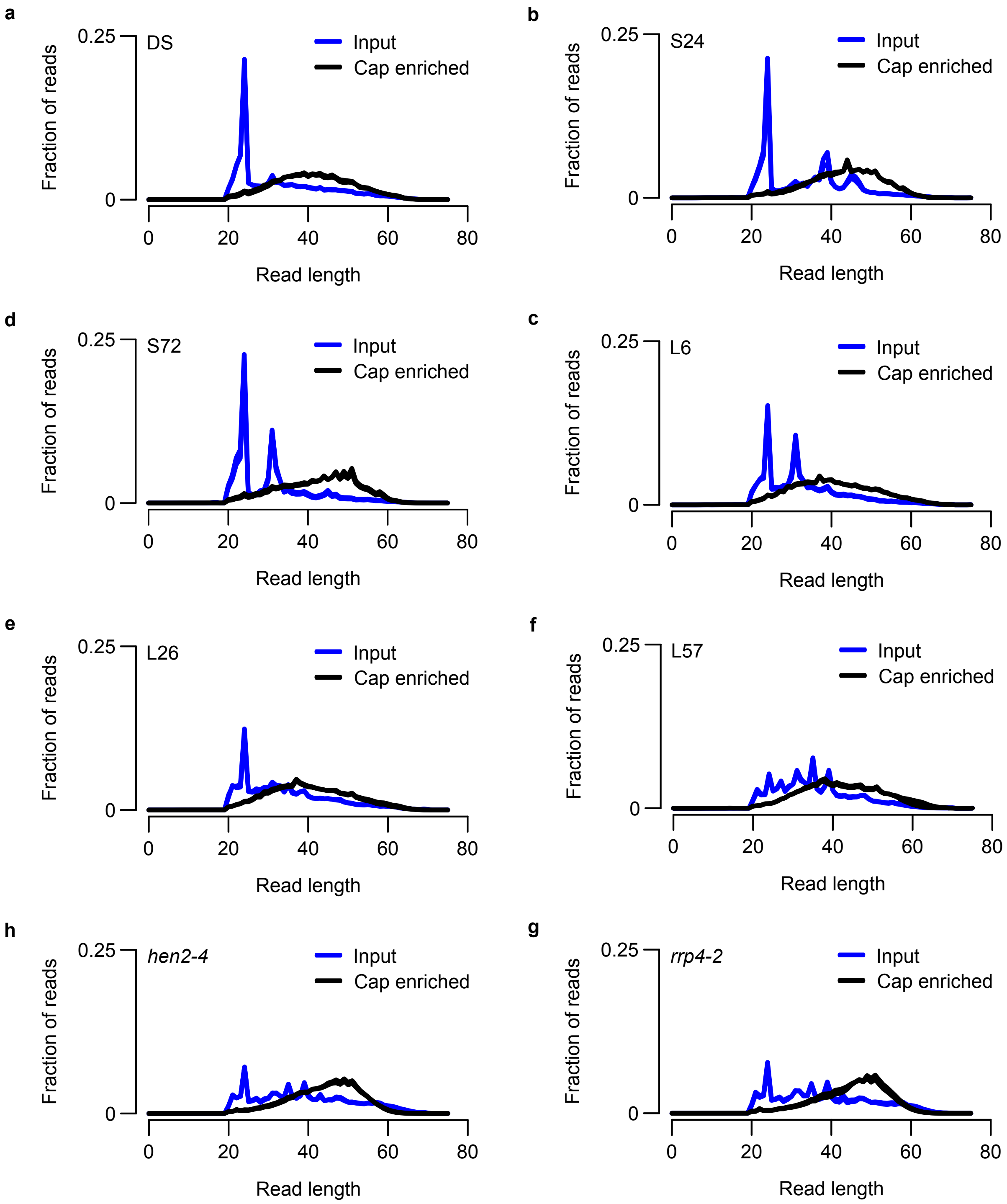

Supplementary Figure 12: csRNA-seq and sRNA-seq read size densities.
(a) - (g) Distribution of filtered read sizes in the sRNA-seq and corresponding csRNA-seq samples.

The csRNA-seq captures transcription initiation independent of transcript stability

Due to the generally inherently unstable nature of non-coding RNAs, these are generally captured in lower abundances in typical RNA-seq experiments. Since our aim with the csRNA-seq was to faithfully capture the levels of genome-wide transcription initiation irrespective of transcript stability during our time-course, we wished to test whether we could observe an under-enrichment of such RNAs in our csRNA-seq datasets. To do this, we repeated the csRNA-seq and RNA-seq experiments using *hen2-4* and *rrp4-2* mutant plants, which accumulate higher levels of unstable non-coding RNAs due to defects in their RNA degradation pathways. Using the non-coding transcriptome data we obtained from these samples, we examined whether these could inform us as to the contribution of cytoplasmic RNAs (as opposed to nascently transcribed RNAs) to the csRNA-seq quantification. Using DE analysis, we found that 13% and 15% of all non-coding TSSs were up-regulated in the two exosome mutants, as well as 18% and 15% of the actual lncRNA transcripts (the majority of detected lncRNAs were up-regulated in the RNA-seq of the exosome mutants, though had insufficiently low P-values due to their low expression), whereas very few were down-regulated in either the csRNA-seq or RNA-seq (Supplementary Figure 13a-d). The data matched the increased quantification of unstable RNA species observed in CAGE data of these mutants, leading us to initially believe the csRNA-seq may be capturing both nascent and cytoplasmic RNAs (Thieffry et al., 2020). However, after assembling a consensus set of up-regulated non-coding TSSs and lncRNAs, we did not observe a significant overlap between the csRNA-seq and RNA-seq data (Supplementary Figure 13e). This led us to conclude that the up-regulation of a subset of non-coding TSSs in the exosome mutants may be as a result of a different mechanism than increased contribution from additional accumulated cytoplasmic RNA. Indeed, while exosome-insensitivity (i.e. no increased stability in the exosome mutants) of lncRNAs was found to be associated with an increase in GC-content, the opposite was true for up-regulated non-coding TSSs in the exosome mutant csRNA-seq samples, with a sharp increase in GC content in a small region immediately downstream of the TSS (which is likely not as a result of uneven library GC content between the L57 and exosome mutant samples; Supplementary Figure 13f-h). We concluded that this could represent an increase in spurious transcription initiation in the exosome-mutants, which may not necessarily lead to productive elongation and that the csRNA-seq quantification of non-coding TSSs in our Col-0 is likely independent of transcript stability. In conclusion, the lack of consensus between the RNA-seq and csRNA-seq expression levels of those non-coding RNAs we found to be unstable suggest there is no association between transcript stability and signal abundance in the csRNA-seq.

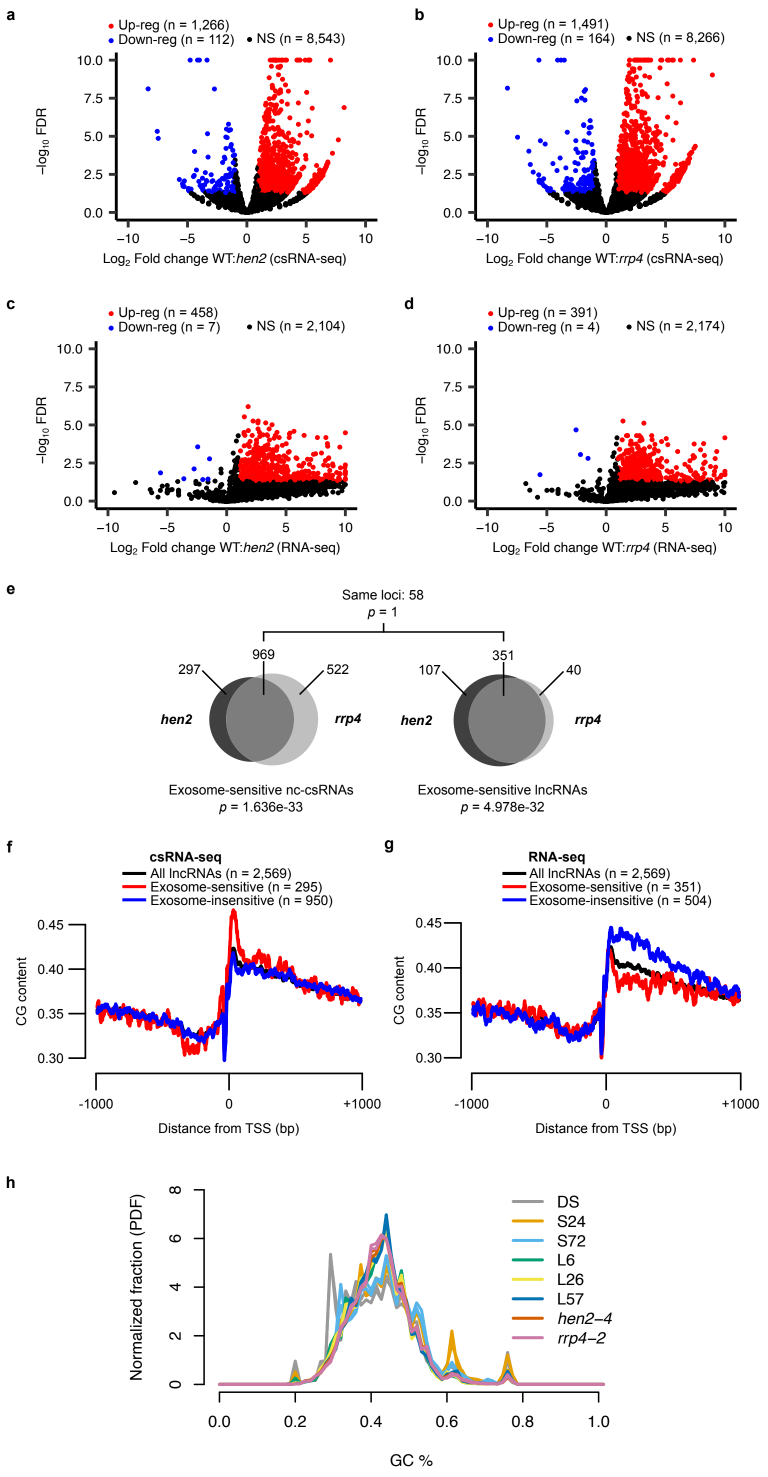

Supplementary Figure 13: Analysis of csRNA-seq and RNA-seq non-coding transcription in *hen2-4* and *rrp4-2*.

(a), (b) Volcano plots of csRNA-seq differential expression of non-coding TSSs between the L57 and exosome mutant samples (*hen2-4* and *rrp4-2*).
(c), (d) Same as (a), (b) for detected lncRNAs using the RNA-seq.
(e) Venn diagrams showing the overlap between significantly upregulated non-coding TSSs and lncRNAs in the *hen2-4* and *rrp4-2* samples compared to L57.
(f), (g) Average GC content in a 2 Kbp window centered around the TSSs of non-coding TSSs and lncRNAs. Also calculated for those classified as exosome-sensitive and insensitive.
(h) Distribution of filtered read GC content in the csRNA-seq samples.

Supplementary Note 2

RNA degradation products are not a significant source of capped-small RNAs in dry seeds

It is generally understood that dry seeds do not undergo active transcriptional elongation as a consequence of their metabolically inert state. Despite this, previous studies have shown that they retain some level of transcriptional competence via the presence of RNAPII in the nucleus (Comai & Harada, 1990; Zhao et al., 2022). As a result, it is logical to conclude that capped-small RNAs (which are the product of RNAPII transcription initiation) would be present within dry seeds, even if they are not being actively elongated. To test this, we examined the read distribution in TSSs and gene bodies in all csRNA-seq samples for evidence of increased RNA degradation products which could suggest a lack of RNAPII transcription initiation-specific products. We first calculated the ratio of reads within genes which were present specifically near the TSS to the entire gene and found that nearly all reads in all csRNA-seq libraries were present within the TSS region (Supplementary Figure 14a), indicating successful enrichment of capped-small RNAs. Repeating the analysis with the input small RNA libraries showed that most detected small RNAs present within gene bodies were not originating from the TSS, though there was increased variability across time-points (Supplementary Figure 14b). Crucially, the dry seed samples did not indicate increased ratios of reads present in the TSS relative to other samples, suggesting these samples did not have a specific increase in TSS-specific degradation products which could generate additional false-positive TSS peaks. We also compared these read counts to their total library sizes and observed largely similar patterns, with typical levels of relative read counts within the TSS regions in both capped-small and input RNA dry seed libraries (Supplementary Figure 14c, d).

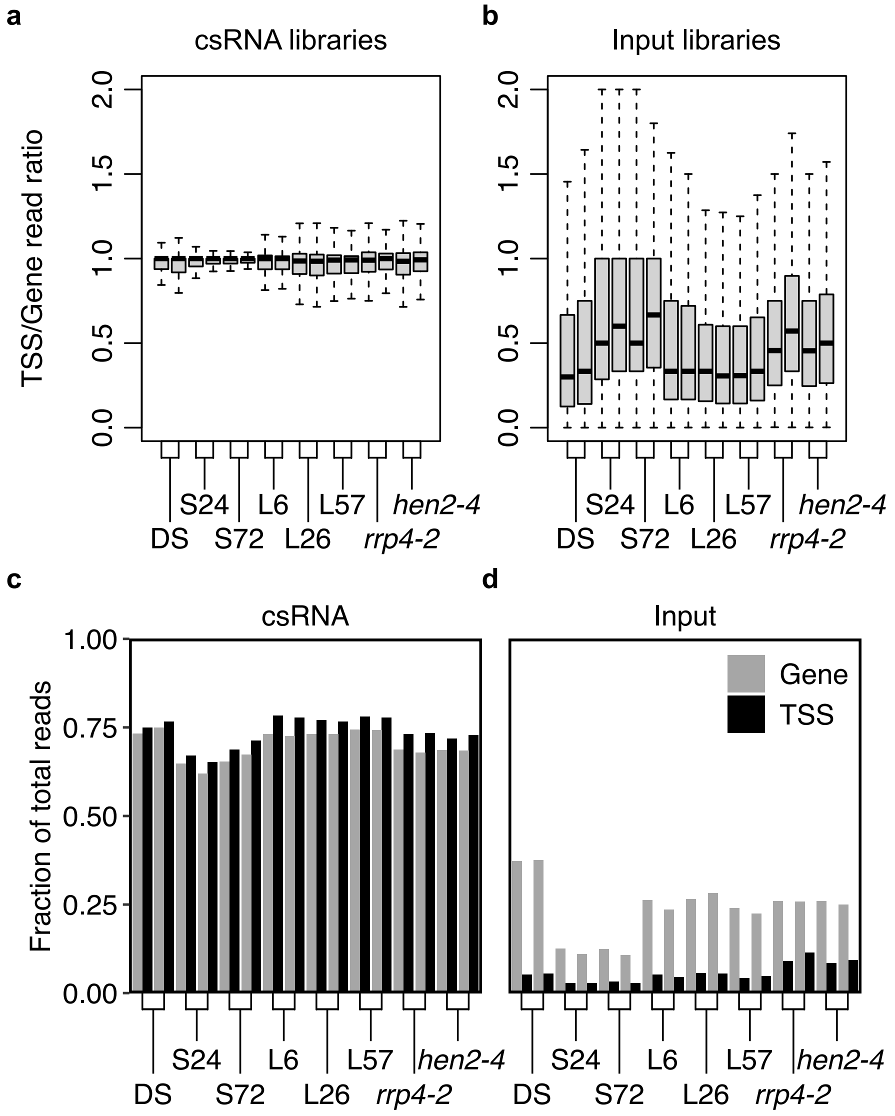

Supplementary Figure 14: Relative csRNA-seq read abundance in TSSs and gene bodies.

(a), (b) Boxplots of the ratio of reads present in the TSS region of genes to the entire gene region in the capped-small and input RNA-seq libraries (n = 19,688). A value of 1 indicates that all reads over a gene are present within the TSS region. Some TSSs overlap regions outside of the gene and thus some ratios are greater than 1. The lower, middle and upper hinges correspond to first quartile, median, and third quartile, respectively. The lower and upper whiskers extend to the minimal/maximal value respectively or 1.5 times the interquartile range, whichever is closer to the median.

(c), (d) Barplots of the fraction of total reads in the capped-small and input RNA-seq libraries present within TSS and gene regions (n = 19,688). The lower, middle and upper hinges correspond to first quartile, median, and third quartile, respectively. The lower and

upper whiskers extend to the minimal/maximal value respectively or 1.5 times the interquartile range, whichever is closer to the median.

RNAPII is present over gene bodies in seeds

To validate the presence of RNAPII over genes within seeds we performed RNAPII ChIP-qPCR targeting *ACT7* and *DOG1* in both dry and imbibed seeds. In both cases we observed significant enrichment of RNAPII near the TSS of each gene when compared to background levels in the genome (Supplementary Figure 15a, b), demonstrating that RNAPII is present in the expected location within genes to have generated initiated capped RNAs of the appropriate size to be enriched in the csRNA-seq.

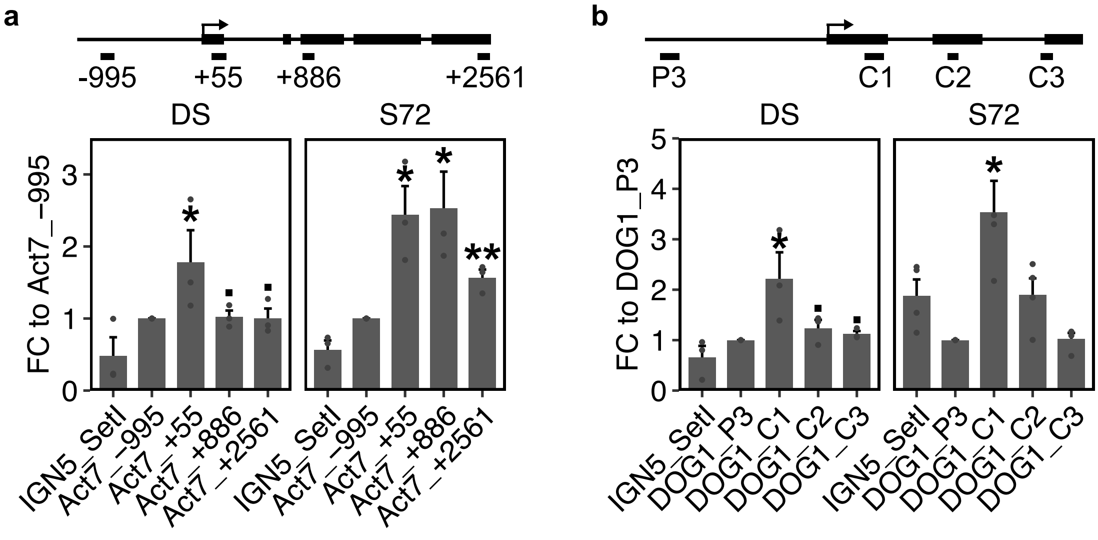

Supplementary Figure 15: RNAPII ChIP-qPCR in dry and imbibed seeds.
(a) RNAPII ChIP-qPCR of dry (DS, n = 3) and imbibed (72 h stratified, S72, n = 3) seeds using primers targeting the *ACTIN7* gene (*Act7*; *AT5G09810*) obtained from (Wu et al., 2016). Input-normalized RNAPII enrichment levels for each sample were normalized to enrichment levels over the promoter of *Act7* (Act7_-995). Statistical significant enrichment of RNAPII over background levels was determined by comparing the enrichment values with those obtained from primers targeting Intergenic Region 5 (IGN5, IGN5_SetI) obtained from (Wu et al., 2016), and performing a one-sided Student's T-test (.P<0.1, *P<0.05, **P<0.01). Error bars indicate the SEM.
(b) RNAPII ChIP-qPCR of dry (DS, n = 3) and imbibed (72 h stratified, S72, n = 4) seeds using primers targeting the *DOG1* gene (*AT5G45830*) obtained from (Chen et al., 2020). Statistical enrichment of RNAPII over background levels was determined as done for (a).

Highly unstable TSS initiation events occur at all stages of germination

Examining the TSSs present within the dry seed-specific cluster in Figure 2a (cluster C1, n = 4,607), 284 are "unstable" TSSs with no detectable RNA-seq signal in any of

our time-points, including the exosome mutants. This suggests these TSSs are sites of RNAPII transcription initiation producing RNAs so unstable they never accumulate to detectable levels during seed maturation. This may not be evidence that they are still being actively initiated in the dry seed, but we believe that at the very least it signifies RNAPII is physically present over these loci at the time of RNA extraction of these samples. Interestingly, expanding this analysis to all clusters reveals a trend whereby the count of unstable TSSs increases dramatically in the C5 and C6 clusters (i.e., the L26 and L57 time-points). This may be indicative of a sharp increase in total transcription initiation upon the transition to post-germinative growth.

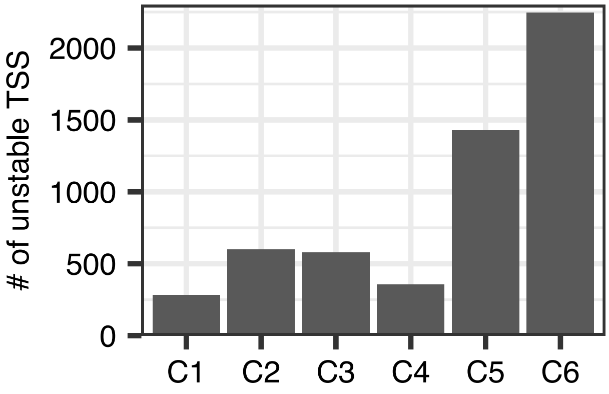

Supplementary Figure 16: Stage-specific Unstable TSSs are detected in all time-points

Tabulation of the number of TSSs without any associated existing transcript annotation or detectable RNA-seq transcript by csRNA-seq cluster (Figure 2a).

Supplementary References

Chen, C. et al. Cytosolic acetyl-CoA promotes histone acetylation predominantly at H3K27 in Arabidopsis. (2017). *Nat. Plants*, *3*, 814–824.

Chen, N., Wang, H., Abdelmageed, H., Veerappan, V., Tadege, M., & Allen, R. D. (2020). HSI2/VAL1 and HSL1/VAL2 function redundantly to repress DOG1 expression in Arabidopsis seeds and seedlings. *New Phytol.*, *227*(3), 840–856. https://doi.org/10.1111/nph.16559

Comai, L., & Harada, J. J. (1990). Transcriptional activities in dry seed nuclei indicate the timing of the transition from embryogeny to germination. *Proceedings of the National Academy of Sciences*, *87*(7), 2671–2674. https://doi.org/10.1073/pnas.87.7.2671

Fedak, H. et al. (2016). Control of seed dormancy in Arabidopsis by a cis-acting noncoding antisense transcript. *Proceedings of the National Academy of Sciences*, *113*, E7846–E7855.

Hetzel, J., Duttke, S. H., Benner, C. & Chory, J. Nascent RNA sequencing reveals distinct features in plant transcription (2016). *Proceedings of the National Academy of Sciences, 113*, 12316–12321.

Inagaki, S., Takahashi, M., Takashima, K., Oya, S. & Kakutani, T. (2021). Chromatin-based mechanisms to coordinate convergent overlapping transcription. *Nat Plants*, *7*, 295–302.

Kang, Y. J. et al. (2017). CPC2: A fast and accurate coding potential calculator based on sequence intrinsic features. *Nucleic Acids Res*, *45*, W12–W16.

Kindgren, P., Ivanov, M. & Marquardt, S. (2020). Native elongation transcript sequencing reveals temperature dependent dynamics of nascent RNAPII transcription in Arabidopsis. *Nucleic Acids Res.*, *48*, 2332–2347.

Li, L. et al. (2021). Global profiling of RNA–chromatin interactions reveals co-regulatory gene expression networks in Arabidopsis. *Nat. Plants*, *7*, 1364–1378.

Mallory, A. C., & Vaucheret, H. (2006). Functions of microRNAs and related small RNAs in plants. *Nature Genetics*, *38*(6), Article 6. https://doi.org/10.1038/ng1791

Thieffry, A. et al. (2022). PAMP-triggered genetic reprogramming involves widespread alternative transcription initiation and an immediate transcription factor wave. *Plant Cell*, *34*, 2615–2637.

Thieffry, A., Vigh, M. L., Bornholdt, J., Ivanov, M., Brodersen, P., & Sandelin, A. (2020). Characterization of arabidopsis thaliana promoter bidirectionality and antisense RNAs by inactivation of nuclear RNA decay pathways. *Plant Cell*, *32*(6), 1845–1867. https://doi.org/10.1105/tpc.19.00815

Tian, F., Yang, D. C., Meng, Y. Q., Jin, J. & Gao, G. (2020). PlantRegMap: Charting functional regulatory maps in plants. *Nucleic Acids Res*, *48*, D1104–D1113.

Wollmann, H. et al. (2017). The histone H3 variant H3.3 regulates gene body DNA methylation in Arabidopsis thaliana. *Genome Biol.*, *18*, 94.

Wu, Z., Ietswaart, R., Liu, F., Yang, H., Howard, M., & Dean, C. (2016). Quantitative regulation of FLC via coordinated transcriptional initiation and elongation. *Proceedings of the National Academy of Sciences of the United States of America*, *113*(1), 218–223. https://doi.org/10.1073/pnas.1518369112

Yadav, V. K., Santos-González, J. & Köhler, C. (2021). INT-Hi-C reveals distinct

chromatin architecture in endosperm and leaf tissues of Arabidopsis. *Nucleic Acids Res*., *49*, 4371–4385.
Yan, W. et al. (2019). Dynamic control of enhancer activity drives stage-specific gene expression during flower morphogenesis. *Nat Commun*, *10*, 1–16.
Zhang, T., Marand, A. P. & Jiang, J. (2016). PlantDHS: a database for DNase I hypersensitive sites in plants. *Nucleic Acids Res*., *44*, D1148–D1153.
Zhao, T., Lu, J., Zhang, H., Xue, M., Pan, J., Ma, L., Berger, F., & Jiang, D. (2022). Histone H3.3 deposition in seed is essential for the post-embryonic developmental competence in Arabidopsis. *Nature Communications*, *13*(1), Article 1. https://doi.org/10.1038/s41467-022-35509-6
Zhu, B., Zhang, W., Zhang, T., Liu, B. & Jiang, J. (2015). Genome-wide prediction and validation of intergenic enhancers in arabidopsis using open chromatin signatures. *Plant Cell*, *27*, 2415–2426.
