# Supplementary material for: Interplay between coding and non-coding regulation drives the Arabidopsis seed-to-seedling transition
Source: Nat Commun. 2024 Feb 26;15:1724. doi: 10.1038/s41467-024-46082-5 (PMC10897432; doi:10.1038/s41467-024-46082-5)
Supplement: Supplementary file 3 — Description of Additional Supplementary Files [file 41467_2024_46082_MOESM3_ESM.pdf]

Description of Additional Supplementary Files:

Supplementary Dataset 1: TSS annotation and quantification.

Supplementary Dataset 2: ACR annotation and quantification.

Supplementary Dataset 3: csRNA-seq differential expression analyses.

Supplementary Dataset 4: csRNA-seq clustering and GO enrichment.

Supplementary Dataset 5: RNA-seq clustering and GO enrichment.

Supplementary Dataset 6: ATAC-seq clustering and GO enrichment.

Supplementary Dataset 7: TomTom similarity between de novo motifs and known transcription factor binding sites.

Supplementary Dataset 8: Protein coding genes with detected antisense TSSs in the csRNA-seq.

Supplementary Dataset 9: Detected bidirectional promoters in the csRNA-seq.

Supplementary Dataset 10: Detected enhancers and their activity during the seedto-seedling transition.
