# Supplementary material for: Interplay between coding and non-coding regulation drives the Arabidopsis seed-to-seedling transition
Source: Nat Commun. 2024 Feb 26;15:1724. doi: 10.1038/s41467-024-46082-5 (PMC10897432; doi:10.1038/s41467-024-46082-5)
Supplement: Supplementary file 14 — Reporting Summary [file 41467_2024_46082_MOESM14_ESM.pdf]

nature portfolio

Corresponding author(s): Julia I. Qüesta

Last updated by author(s): Feb 2, 2024

Reporting Summary

Nature Portfolio wishes to improve the reproducibility of the work that we publish. This form provides structure for consistency and transparency in reporting. For further information on Nature Portfolio policies, see our Editorial Policies and the Editorial Policy Checklist.

Statistics

For all statistical analyses, confirm that the following items are present in the figure legend, table legend, main text, or Methods section.

n/a	Confirmed	
☐	☒	The exact sample size (*n*) for each experimental group/condition, given as a discrete number and unit of measurement
☐	☒	A statement on whether measurements were taken from distinct samples or whether the same sample was measured repeatedly
☐	☒	The statistical test(s) used AND whether they are one- or two-sided<br>*Only common tests should be described solely by name; describe more complex techniques in the Methods section.*
☒	☐	A description of all covariates tested
☐	☒	A description of any assumptions or corrections, such as tests of normality and adjustment for multiple comparisons
☐	☒	A full description of the statistical parameters including central tendency (e.g. means) or other basic estimates (e.g. regression coefficient) AND variation (e.g. standard deviation) or associated estimates of uncertainty (e.g. confidence intervals)
☒	☐	For null hypothesis testing, the test statistic (e.g. *F*, *t*, *r*) with confidence intervals, effect sizes, degrees of freedom and *P* value noted<br>*Give P values as exact values whenever suitable.*
☒	☐	For Bayesian analysis, information on the choice of priors and Markov chain Monte Carlo settings
☒	☐	For hierarchical and complex designs, identification of the appropriate level for tests and full reporting of outcomes
☐	☒	Estimates of effect sizes (e.g. Cohen's *d*, Pearson's *r*), indicating how they were calculated

Our web collection on statistics for biologists contains articles on many of the points above.

Software and code

Policy information about availability of computer code

Data collection	qPCR data collection was performed using the LightCycler 480 Software (version 1.5.1.62 SP3).
Data analysis	HOMER (version 4.11.1)<br>STAR (version 2.7.9a)<br>samtools (version 1.14)<br>R (version 4.3.1)<br>GFF3sort (version 1.0.0)<br>stringtie (version 2.1.4)<br>fastp (version 0.23.4)<br>bowtie2 (version 2.4.4)<br>MACS3 (version 3.0.0b3)<br>bedtools (version 2.30.0)<br>bigWigAverageOverBed (version 2)<br>streme (version 5.4.1)<br>tomtom (version 5.4.1)<br>sea (version 5.4.1)<br>Genrich (version 0.6.1)<br>gffcompare (version 0.12.6)

gffread (version 0.12.7)
awk (version 20200816)
python (version 3.11.4)

For manuscripts utilizing custom algorithms or software that are central to the research but not yet described in published literature, software must be made available to editors and reviewers. We strongly encourage code deposition in a community repository (e.g. GitHub). See the Nature Portfolio guidelines for submitting code & software for further information.

Data

Policy information about availability of data

All manuscripts must include a data availability statement. This statement should provide the following information, where applicable:

- Accession codes, unique identifiers, or web links for publicly available datasets
- A description of any restrictions on data availability
- For clinical datasets or third party data, please ensure that the statement adheres to our policy

All csRNA-seq, sRNA-seq, RNA-seq and ATAC-seq datasets generated in this study are available from the NCBI GEO repository under the accession GSE250331. Original code and data can be accessed from https://github.com/bjmt/Tremblay_et_al_2024_Seed_to_seedling. Plasmids and mutant lines generated in this study are available upon reasonable request to the corresponding author. Source data are provided with this paper.

Research involving human participants, their data, or biological material

Policy information about studies with human participants or human data. See also policy information about sex, gender (identity/presentation), and sexual orientation and race, ethnicity and racism.

Reporting on sex and gender	*Use the terms sex (biological attribute) and gender (shaped by social and cultural circumstances) carefully in order to avoid confusing both terms. Indicate if findings apply to only one sex or gender; describe whether sex and gender were considered in study design; whether sex and/or gender was determined based on self-reporting or assigned and methods used. Provide in the source data disaggregated sex and gender data, where this information has been collected, and if consent has been obtained for sharing of individual-level data; provide overall numbers in this Reporting Summary. Please state if this information has not been collected. Report sex- and gender-based analyses where performed, justify reasons for lack of sex- and gender-based analysis.*
Reporting on race, ethnicity, or other socially relevant groupings	*Please specify the socially constructed or socially relevant categorization variable(s) used in your manuscript and explain why they were used. Please note that such variables should not be used as proxies for other socially constructed/relevant variables (for example, race or ethnicity should not be used as a proxy for socioeconomic status). Provide clear definitions of the relevant terms used, how they were provided (by the participants/respondents, the researchers, or third parties), and the method(s) used to classify people into the different categories (e.g. self-report, census or administrative data, social media data, etc.) Please provide details about how you controlled for confounding variables in your analyses.*
Population characteristics	*Describe the covariate-relevant population characteristics of the human research participants (e.g. age, genotypic information, past and current diagnosis and treatment categories). If you filled out the behavioural & social sciences study design questions and have nothing to add here, write "See above."*
Recruitment	*Describe how participants were recruited. Outline any potential self-selection bias or other biases that may be present and how these are likely to impact results.*
Ethics oversight	*Identify the organization(s) that approved the study protocol.*

Note that full information on the approval of the study protocol must also be provided in the manuscript.

Field-specific reporting

Please select the one below that is the best fit for your research. If you are not sure, read the appropriate sections before making your selection.

[x] Life sciences [ ] Behavioural & social sciences [ ] Ecological, evolutionary & environmental sciences

For a reference copy of the document with all sections, see nature.com/documents/nr-reporting-summary-flat.pdf

Life sciences study design

All studies must disclose on these points even when the disclosure is negative.

Sample size	No sample size calculations were performed. Sample sizes were chosen based on available material and the minimum number required for statistical testing.
Data exclusions	No data were excluded.
Replication	Biological replicates were used for all experiments. For sequencing experiments, inter-replicate reproducibility was evaluated using Pearson Correlation coefficients. For analysis of mutant lines, two individual mutant lines were tested for each gene of interest. For ChIP-qPCR, two

individual genes were tested for each sample.

Randomization	Not relevant to this study.
Blinding	Not relevant to this study.

Reporting for specific materials, systems and methods

We require information from authors about some types of materials, experimental systems and methods used in many studies. Here, indicate whether each material, system or method listed is relevant to your study. If you are not sure if a list item applies to your research, read the appropriate section before selecting a response.

Materials & experimental systems

n/a	Involved in the study
☐	☒ Antibodies
☒	☐ Eukaryotic cell lines
☒	☐ Palaeontology and archaeology
☒	☐ Animals and other organisms
☒	☐ Clinical data
☒	☐ Dual use research of concern
☐	☒ Plants

Methods

n/a	Involved in the study
☒	☐ ChIP-seq
☒	☐ Flow cytometry
☒	☐ MRI-based neuroimaging

Antibodies

Antibodies used	Pol II Antibody (Diagenode Cat# C15200004 Lot# 001-13)
Validation	Validation of the antibody was peformed by Diagenode for use in human HeLa cells with ChIP-qPCR and use in human HeLaS3 cells with ChIP-seq. Antibody specificity was tested by Diagenode using an ELISA against peptides containing the unmodified C-terminal repeat sequence as well as different phosphorylated peptides. (https://www.diagenode.com/en/p/pol-ii-monoclonal-antibody-classic-50-mg)<br><br>This antibody has been used succesfully for ChIP-seq in Arabidopsis thaliana by Inagaki et al. Nat Plants 2021 (10.1038/s41477-021-00868-3).

Plants

Seed stocks	Arabidopsis thaliana seeds were obtained from the Nottingham Arabidopsis Stock Centre: Col-0 seed, SAIL_1250_D04 (NASC ID: N846065), SALK_201027C (NASC ID: N688329), SALK_138567 (NASC ID: N887863), SALK_073206 (NASC ID: N573206). hen2-4 and rrp4-2 seed were provided as gifts by Heike Lange and Peter Brodersen, respectively.
Novel plant genotypes	Two independent mutant lines deleting the bidirectional promoter region of SLP2 were generated using CRISPR-Cas9. The Golden Gate method was used to construct a vector expressing two guide RNAs (gRNAs) that target SLP2 enhancer region to generate deletions. gRNAs were designed using the following web tool: http://crispr.hzau.edu.cn/cgi-bin/CRISPR2/CRISPR. For assembly, the two gRNA sequences were incorporated into PCR forward and reverse primers (Table S1). The PCR fragment was amplified from pCBC-DT1T2, purified and inserted into pHEE401E binary vector (carrying CAS9 construct) by Golden Gate reaction. The binary vector obtained was transformed into A. tumefaciens GV3010 strain and subsequently transformed into Arabidopsis Col-0 by the floral dip method. All experiments were performed using seed from homozygous T3 plants.
Authentication	All mutant lines were genotyped using PCR with the primers indicated in Table S1. Independent lines were used for all experiments to offset the effects of secondary mutations.
